# Mechanistic insights into the key marine dimethylsulfoniopropionate synthesis enzyme DsyB/DSYB

**DOI:** 10.1002/mlf2.12030

**Published:** 2022-06-15

**Authors:** Chun‐Yang Li, Jason C. Crack, Simone Newton‐Payne, Andrew R. J. Murphy, Xiu‐Lan Chen, Benjamin J. Pinchbeck, Shun Zhou, Beth T. Williams, Ming Peng, Xiao‐Hua Zhang, Yin Chen, Nick E. Le Brun, Jonathan D. Todd, Yu‐Zhong Zhang

**Affiliations:** ^1^ Frontiers Science Center for Deep Ocean Multispheres and Earth System, College of Marine Life Sciences Ocean University of China Qingdao China; ^2^ State Key Laboratory of Microbial Technology Marine Biotechnology Research Center, Shandong University Qingdao China; ^3^ Laboratory for Marine Biology and Biotechnology Pilot National Laboratory for Marine Science and Technology Qingdao Shandong China; ^4^ School of Chemistry, Centre for Molecular and Structural Biochemistry University of East Anglia, Norwich Research Park Norwich UK; ^5^ School of Biological Sciences University of East Anglia Norwich UK; ^6^ School of Life Sciences University of Warwick Coventry UK

**Keywords:** catalytic mechanism, DMSP synthesis, marine sulfur cycle, *S*‐methyltransferase

## Abstract

Marine algae and bacteria produce approximately eight billion tonnes of the organosulfur molecule dimethylsulfoniopropionate (DMSP) in Earth's surface oceans annually. DMSP is an antistress compound and, once released into the environment, a major nutrient, signaling molecule, and source of climate‐active gases. The methionine transamination pathway for DMSP synthesis is used by most known DMSP‐producing algae and bacteria. The *S*‐directed *S*‐adenosylmethionine (SAM)‐dependent 4‐methylthio‐2‐hydroxybutyrate (MTHB) *S*‐methyltransferase, encoded by the *dsyB/DSYB* gene, is the key enzyme of this pathway, generating *S*‐adenosylhomocysteine (SAH) and 4‐dimethylsulfonio‐2‐hydroxybutyrate (DMSHB). *DsyB*/*DSYB*, present in most haptophyte and dinoflagellate algae with the highest known intracellular DMSP concentrations, is shown to be far more abundant and transcribed in marine environments than any other known *S*‐methyltransferase gene in DMSP synthesis pathways. Furthermore, we demonstrate *in vitro* activity of the bacterial DsyB enzyme from *Nisaea denitrificans* and provide its crystal structure in complex with SAM and SAH‐MTHB, which together provide the first important mechanistic insights into a DMSP synthesis enzyme. Structural and mutational analyses imply that DsyB adopts a proximity and desolvation mechanism for the methyl transfer reaction. Sequence analysis suggests that this mechanism may be common to all bacterial DsyB enzymes and also, importantly, eukaryotic DSYB enzymes from e.g., algae that are the major DMSP producers in Earth's surface oceans.

## Impact statement

Dimethylsulfoniopropionate (DMSP) is one of Earth's most abundant organosulfur‐containing molecules which influence marine nutrient cycling, chemotaxis, atmospheric chemistry, and potentially the climate. This study provides the first structural and mechanistic understanding of the key DMSP synthesis enzyme in marine bacteria (DsyB) and algae (DSYB) that are responsible for the annual production of ~8 billion tonnes of DMSP. DsyB is the first DMSP synthesis enzyme to be analyzed at the structural and mechanistic level. This study defines the functional characteristics of the DsyB/DSYB enzyme family that has a central role in driving the marine microbial cycling of organosulfur.

## INTRODUCTION

Approximately eight billion tonnes of the compatible solute dimethylsulfoniopropionate (DMSP) are produced annually in Earth's surface waters^1^, constituting up to 10% of surface ocean organic carbon^2^. Many marine algae, bacteria, corals, and some plants produce DMSP^3^ for its proposed functions as for example, a compatible solute^4^, grazing deterrent^5^, antioxidant^6^, and protectant against hydrostatic pressure^7^. Furthermore, DMSP is a major nutrient for marine microorganisms, and a precursor for climate‐active volatiles such as dimethyl sulfide (DMS)^3^, ^8^, ^9^. DMSP was thought to be mainly produced by marine algae in Earth's surface oceans, but recent studies suggest that bacteria, particularly in marine sediment, are also important DMSP producers^3^, ^10^, ^11^, ^12^.

**Figure 1:**
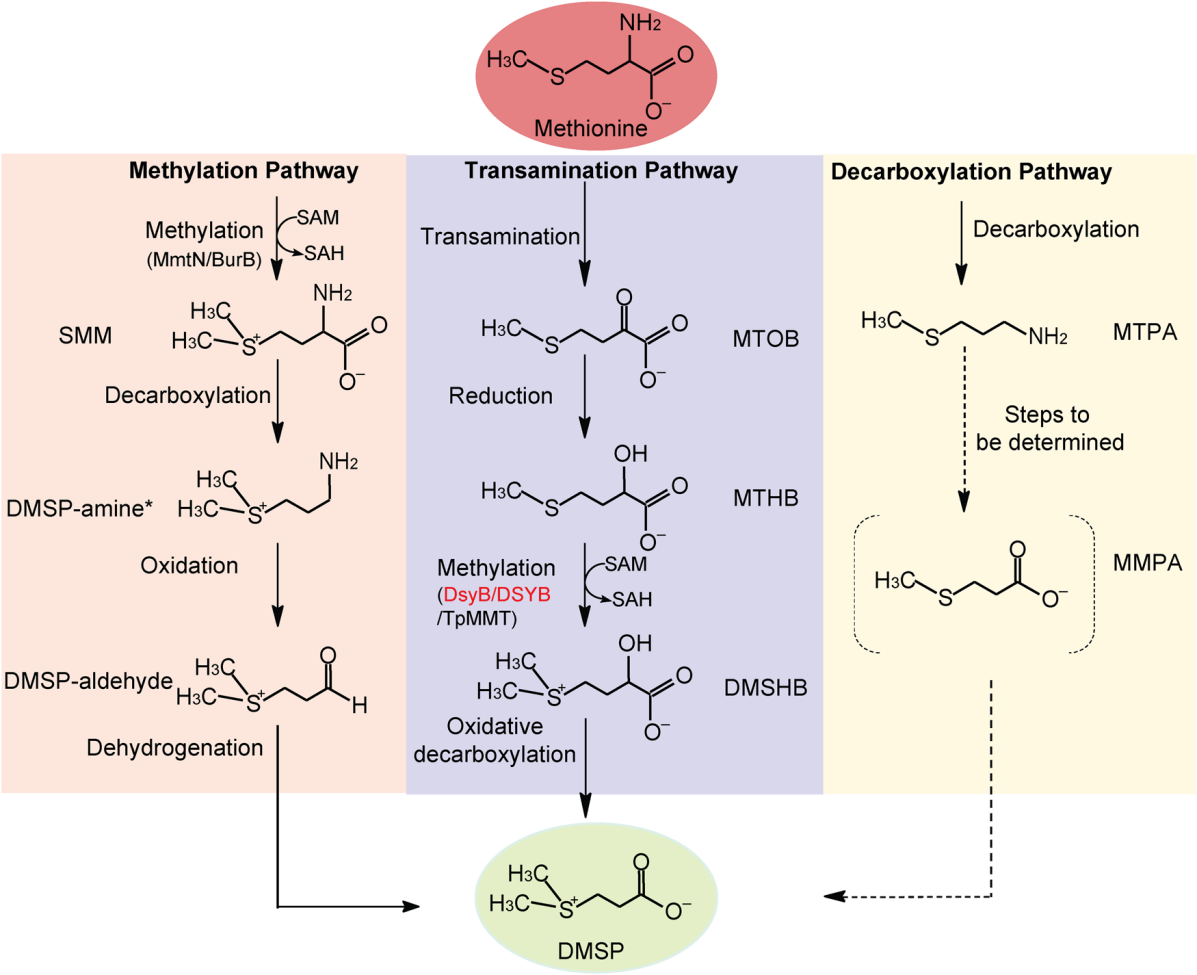
Predicted DMSP biosynthesis pathways^10^. Different pathways are shown in different colors. Enzymes of interest in this study (DsyB/DSYB) are in red. *SMM is converted to DMSP‐aldehyde directly in *Wollastonia*. Dotted lines represent unconfirmed steps of the decarboxylation pathway. DMSHB, 4‐dimethylsulfonio‐2‐hydroxybutyrate; DMSP, dimethylsulfoniopropionate; MMPA, methylmercaptopropionate; MTHB, 4‐methylthio‐2‐hydroxybutyrate; MTOB, 4‐methylthio‐2‐oxobutyrate; MTPA, 3‐methylthiopropylamine; SAH, *S*‐adenosylhomocysteine; SAM, *S*‐adenosylmethionine; SMM, *S*‐methylmethionine.

Three pathways for DMSP synthesis have been proposed based on the identification of intermediates and enzyme activities in various model DMSP producers: a methylation pathway in some plants and bacteria, a transamination pathway in algae and bacteria, and a decarboxylation pathway in onedinoflagellate^10^, ^12^, ^13^, ^14^, ^15^ (Figure 1). Of these, the transamination pathway is thought to be the most important in marine environments as it functions in the majority of DMSP‐producing algae (spanning dinoflagellates, haptophytes, and diatoms) and bacteria^10^, ^12^, ^14^. The committed enzyme of the transamination pathway (Figure 1) is an *S*‐adenosylmethionine (SAM)‐dependent 4‐methylthio‐2‐hydroxybutyrate (MTHB) *S*‐methyltransferase that yields 4‐dimethylsulfonio‐2‐hydroxybutyrate (DMSHB)^13^, ^14^, ^16^. Recently, the key MTHB *S*‐methyltransferase enzyme ‘DsyB’, was identified in many DMSP‐producing marine *Alphaproteobacteria* ^10^. Enzymes, termed DSYB, with ∼33% amino acid identity to bacterial DsyB enzymes and that have SAM‐dependent MTHB*S*‐methyltransferase activity are found in many eukaryotes including most DMSP‐producing dinoflagellates, haptophytes, corals, and ∼20% of diatoms^12^. This is consistent with the detection of DMSHB and its oxidative decarboxylation to DMSP in some prymnesiophytes, diatoms, and prasinophytes^14^. * dsyB*/*DSYB* genes are robust indicators of an organism's potential to produce DMSP^10^, ^12^. The centric diatom *Thalassiosira pseudonana*, which lacks DSYB, contains an isoform MTHB *S*‐methyltransferase enzyme termed TpMMT, but this enzyme has not been studied in any other organism^17^. Published and new analysis here (see below) shows that *DsyB*/*DSYB* genes are far more abundant in known DMSP‐producing microorganisms (phytoplankton and bacteria) and in the marine environmental metagenome and metatranscriptome datasets than other identified DMSP synthesis genes^10^, ^11^, ^12^. Furthermore, acquisition of *dsyB* is sufficient to enable some organisms to produce DMSP^10^. Together, these data suggest that the transamination pathway using DsyB/DSYB enzymes is the most important marine DMSP synthesis pathway.

DSYB and DsyB belong to the SAM‐dependent methyltransferase (SAM‐MT) family^10^, ^12^. SAM is the second most widely used enzyme‐substrate after ATP and is involved in many important biological processes^18^. SAM‐MTs are categorized based on the methyl‐accepting atom, usually *O*, *N*, *C*, or *S* ^19^. The majority (54%) of known SAM‐MTs are *O*‐directed, whereas only 3% are *S*‐directed^19^. SAM‐MTs, which catalyze transmethylation via S_N_2 nucleophilic substitution^20^, ^21^, have evolved three distinct mechanisms: proximity and desolvation (PD), general acid/base‐mediated catalysis, and a metal‐dependent mechanism^19^. There are no reported protein crystallographic studies on any DMSP synthesis enzyme and, thus, the mechanism of *S*‐directed SAM‐MT in DMSP synthesis pathways, for example, via DsyB/DSYB or TpMMT, are unknown.

Here, we investigate *Nisaea denitrificans* DR41_21, a marine *Alphaproteobacterium* ^22^ predicted to produce DMSP, and characterize its DsyB enzyme. X‐ray crystallography and mutational analyses are employed to establish the DsyB structure and predict its interaction with SAM and MTHB substrates and reaction mechanism. Furthermore, sequence alignment and structural analysis are used to infer mechanistic similarities between bacterial DsyB and algal DSYB enzymes. We also probe marine microorganisms, metagenomes, and metatranscriptomes for the presence of DsyB/DSYB and other key SAM‐MT in DMSP synthesis pathways to investigate the importance of these proteins in the global oceans. Our results provide the first insights into the mechanism of global DMSP production via the most abundant known DMSP synthesis enzymes.

## RESULTS AND DISCUSSION

### *N. denitrificans* DR41_21 is a DMSP‐producing bacterium

**Figure 2:**
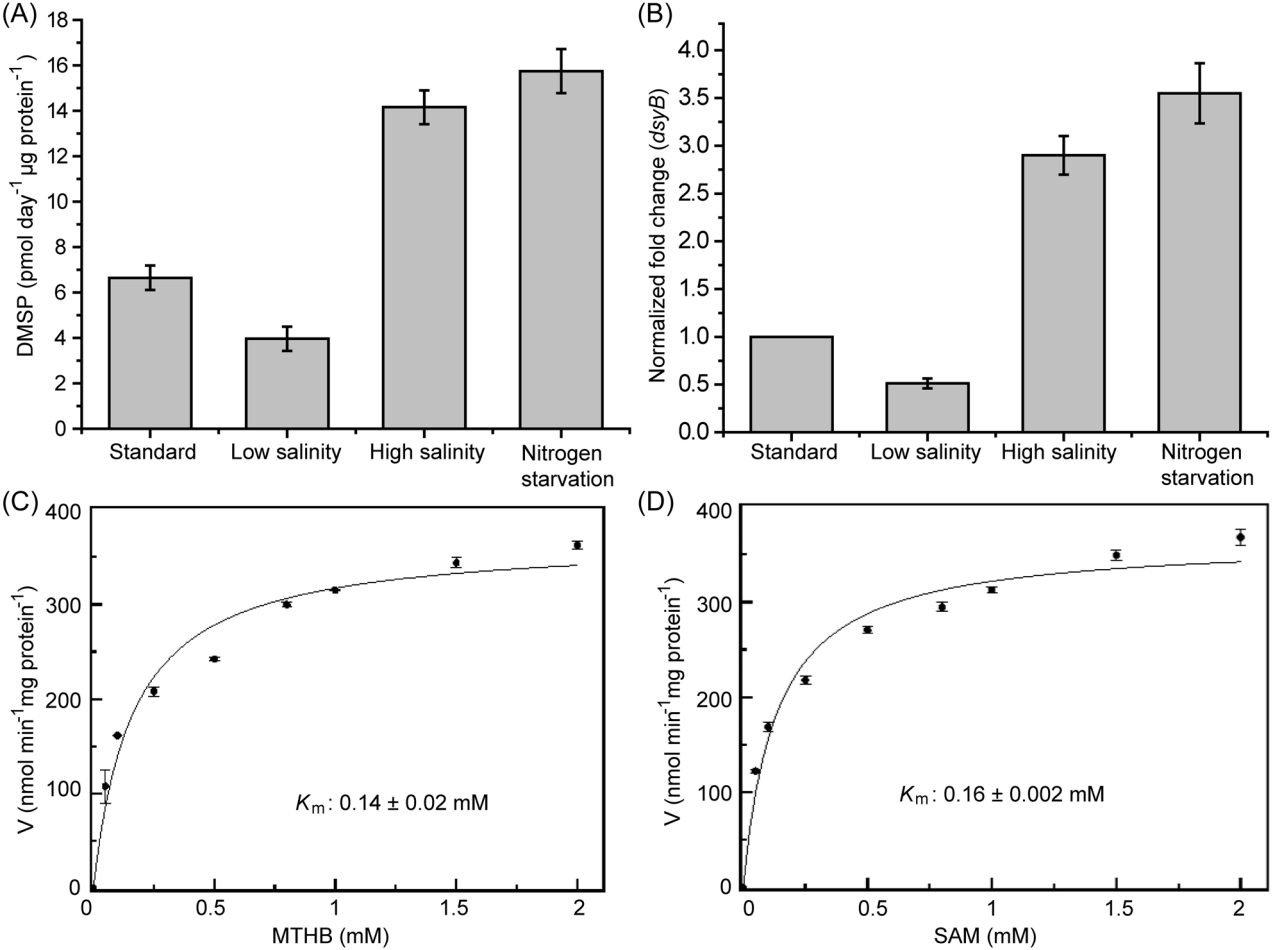
Characterization of *Nisaea dentrificans* DMSP production, *dsyB* transcription, and the DsyB enzyme. *N. dentrificans* DMSP production (A) and *dsyB* transcription (B) observed under different conditions. Standard conditions were MBM medium at 35 PSU with 10 mM NH_4_Cl compared to low salinity (5 PSU), high salinity (50 PSU), and nitrogen starvation conditions (where cells in standard MBM were resuspended in standard MBM with no added NH_4_Cl). (C) A nonlinear fit curve for MTHB methylation by DsyB. Initial rates were determined with 1.97 µM DsyB (50 mM Tris‐HCl, 100 mM NaCl, pH 8.0) and 0–2 mM MTHB. *K*_m_ was 0.14 ± 0.02 mM. (D) A nonlinear fit curve for SAM demethylation by DsyB. Initial rates were determined with 1.97 µM DsyB and 0–2 mM SAM in the same reaction buffer. *K*_m_ was 0.16 ± 0.002 mM. The error bar represents the standard deviation of triplicate experiments. MBM, marine basal minimal; PSU, practical salinity unit.

Isolated from coastal Mediterranean Sea surface waters, *N. denitrificans* DR41_21 (DSM 18348) is a marine *Alphaproteobacterium* of the *Rhodospirillaceae* family which was not previously known to produce DMSP^22^. *N. denitrificans* contains a DsyB enzyme, 337 amino acid residues in length with 59% identity to *Labrenzia aggregata* DsyB and is thus predicted to make DMSP^10^. Indeed, cloned *N. denitrificans dsyB* conferred onto *Rhizobium*, a heterologous host that lacks DsyB and makes no DMSP, MTHB *S*‐methyltransferase activity. Furthermore, *N. denitrificans dsyB* fully restored the production and accumulation of DMSP (105 ± 3.4 pmol DMSP µg protein^−1^) of an *L. aggregata dsyB* ^*−*^ deletion mutant, which produces and accumulates no DMSP (wild‐type *L. aggregata* produces 99.8 ± 1.2 pmol DMSP µg protein^−1^)^10^. *N. denitrificans* itself produced DMSP when grown in the absence of methylated sulfur compounds, and DMSP production and *dsyB* transcription were enhanced by increased salinity and by nitrogen starvation (Figure 2A,B). This study further confirms that the presence of *dsyB* and its transcription in a bacterium reports the ability of the strain to produce DMSP and the levels it makes, respectively.

### *In vitro* characterization of *N. denitrificans* DsyB

As shown above and in Curson et al.^10^, DsyB has MTHB *S*‐methyltransferase activity when expressed in alphaproteobacterial hosts. However, *L. aggregata* DsyB and *Chrysochromulina* DSYB enzymes^12^ had no detectable MTHB *S*‐methyltransferase activity when expressed in *Escherichia coli*. The same was generally true of the recombinant *N. denitrificans* DsyB enzyme purified from *E. coli*, although variable MTHB *S*‐methyltransferase activity was observed *in vitro* using MTHB and SAM as substrates (see Materials and Methods section). The reason for this lack of activity upon isolation is unknown; one possibility is that the enzyme requires an essential co‐factor or modification that was provided by an algal or alphaproteobacterial host but not by *E. coli* ^12^. This hypothesis was initially supported by the fact that the addition of heat‐denatured cell lysate fractions (from a PD10 desalting column) liberated from the *L. aggregata dsyB^−^* deletion mutant, which produces no DMSP, recovered *N. denitrificans* DsyB MTHB *S*‐methyltransferase activity. Similar complementation was shown with the addition of heat‐killed *Prymnesium parvum* extracts to DSYB in ref.^12^. The activated DsyB protein was shown to have *K*_m_ and *V*_max_ values of 0.14 mM and 365 nmol min^−1^ mg protein^−1^, respectively, for MTHB (Figure 2C), which were similar to those previously established for *P. parvum* DSYB (0.09 mM and 294 nmol min^−1^ mg protein^−1^) in Curson et al.^12^ The activated DsyB had a *K*_m_ of 0.16 mM and *V*_max_ 368.9 nmol min^−1^ mg protein^−1^ for the cosubstrate SAM (Figure 2D), which were also similar to those obtained with *P. parvum* DSYB (0.06 mM and 303 nmol min^−1^ mg protein^−1^) in ref.^12^.

Liquid chromatography with mass spectrometry (LC‐MS) and/or native mass spectrometry were used in an attempt to identify the activation factor in the *L. aggregata dsyB* ^*−*^ extracts (Figure S1). A prominent peak at 37,084 Da was observed in the LC‐MS spectrum for both the as‐isolated and activated samples, which corresponds to DsyB with its N‐terminal Met residue cleaved (commonly observed for proteins overexpressed in *E. coli*)^23^. A lower intensity peak at +131 Da, corresponding to the full‐length protein (37,215 Da), was also observed in the as‐isolated sample, indicating that the Met cleavage was not complete (Figure S1A). There was an additional minor peak at +269 Da of unknown origin in the activated sample (Figure S1B). Under nondenaturing conditions, both monomeric and dimeric forms of DsyB were detected in the as‐isolated sample, a feature commonly observed in nondenaturing mass spectra of solution dimers^24^, ^25^, ^26^. In the monomeric region, the main protein peaks (due to cleaved and noncleaved proteins) were again observed, but, in addition, a number of adduct species were present in the spectrum. Two of these, at +36 and +98 Da, correspond to chloride and (most likely) phosphate adducts. An additional adduct at +63 Da was also observed, possibly due to metal ion binding. In general, the spectrum of the dimeric form of DsyB was less well‐resolved, but the main protein peak (at 74,168 Da) along with chloride and possible metal ion adducts were all detected (Figure S2). The non‐denaturing mass spectrum of the activated monomeric DsyB (Figure S1B) revealed a number of adducts, including those most likely due to chloride, phosphate, and metal ion binding (all common within the as‐isolated DsyB), along with an additional adduct at +122 Da (and at +244 Da), which is likely due to Tris buffer. Thus, we have no data to support there being a cofactor or modification of DsyB caused by the addition of the heat‐killed *L. aggregata dsyB* ^*−*^ extracts to as‐isolated DsyB, and further work is required to understand the variable nature of DsyB activity (see below).

The association of metal ions with DsyB was investigated further. Inductively coupled plasma mass spectrometry (ICP‐MS) analysis revealed variable metal ion content with some preparations of as‐isolated DsyB containing up to 0.85 Cu per protein, with other metals such as Ni (up to 0.5 per protein), Zn (0.4) and Fe (0.14) also detected. However, there was no correlation between metal ion content and activity of as‐isolated samples.

Despite mostly lacking consistent *in vitr*o MTHB *S*‐methyltransferase activity (see below), native MS showed that the as‐isolated *N. denitrificans* DsyB enzyme binds to SAM. The deconvoluted mass spectrum of a DsyB sample under nondenaturing conditions and containing 25 equivalents of SAM contained a peak in the DsyB dimer region at +870 Da (predicted mass of a (DsyB)_2_‐(SAM)_2_ is 74,966 Da), indicative of a (DsyB)_2_‐(SAM‐Cl)_2_ adduct, that was not observed in the absence of SAM (Figure S3A). Evidence for SAM binding was also apparent in the monomer region, though the presence of chloride adducts spreads out the intensity in this region (Figure S3B). Evidence for an MTHB‐bound form of DsyB was also observed (although, again, the presence of chloride adducts spreads out intensity; Figure S3B). Although care is needed in interpreting intensities of peaks in the nondenaturing mass spectrum, the low intensity of the SAM‐ and MTHB‐bound forms of DsyB suggests relatively low affinities when these substrates are present individually.

**Figure 3:**
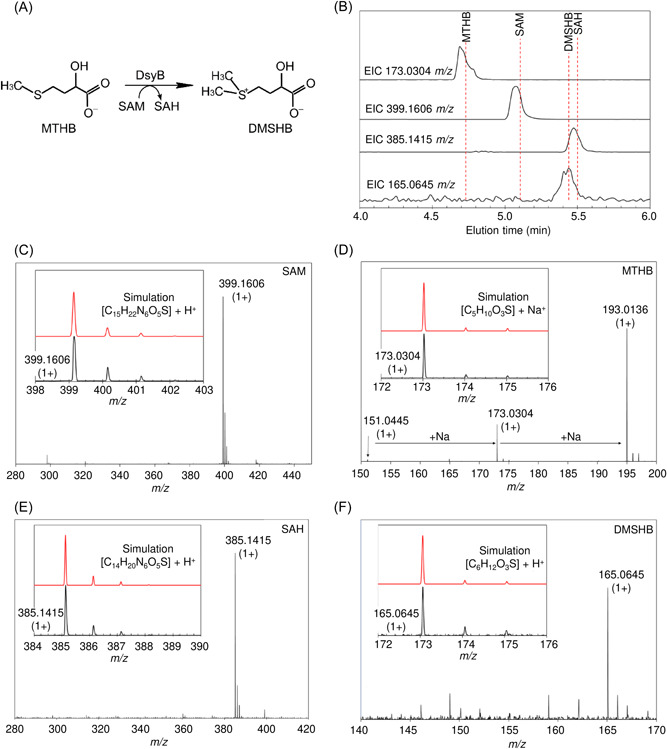
Identification of DsyB substrates and products by LC‐MS. (A) The chemical equation for MTHB *S*‐methylation into DMSHB. (B) Extracted ion chromatograms for MTHB, SAM, DMSHB, and SAH. Mass spectrometry data were analyzed to extract ion counts as a function of elution volume for the *m/z* ions indicated, which correspond to the substrates and products of the DsyB‐catalyzed reaction. The red broken lines indicate elution volumes of the molecules when run as standards. (C–F) Mass spectra recorded for the eluted species, as indicated. Insets are spectra over a narrower *m/z* range (black lines) along with simulated spectra (showing the isotope distribution, red line) for each molecule, providing clear confirmation of identity. LC‐MS, liquid chromatography with mass spectrometry.

Small molecule hydrophilic interaction liquid chromatography (HILIC)‐MS analysis of reactions following the addition of MTHB to DsyB‐SAM resulted in the detection of substrates SAM and MTHB, and products DMSHB and SAH (Figure 3). Nondenaturing mass spectrometry of similarly generated samples resulted in the loss of SAM‐ and MTHB‐bound forms of DsyB (Figure S3B). Together, the data are consistent with DsyB being a SAM‐dependent MTHB *S*‐methyltransferase.

### Overall structure of DsyB

To analyze the catalytic mechanism of DsyB, we solved the crystal structures of complexes of DsyB with SAM and with SAH‐MTHB. The crystal structure of the DsyB‐SAM complex was determined by the single‐wavelength anomalous dispersion (SAD) method using a selenomethionine derivative (Se derivative; Table S1).

**Figure 4:**
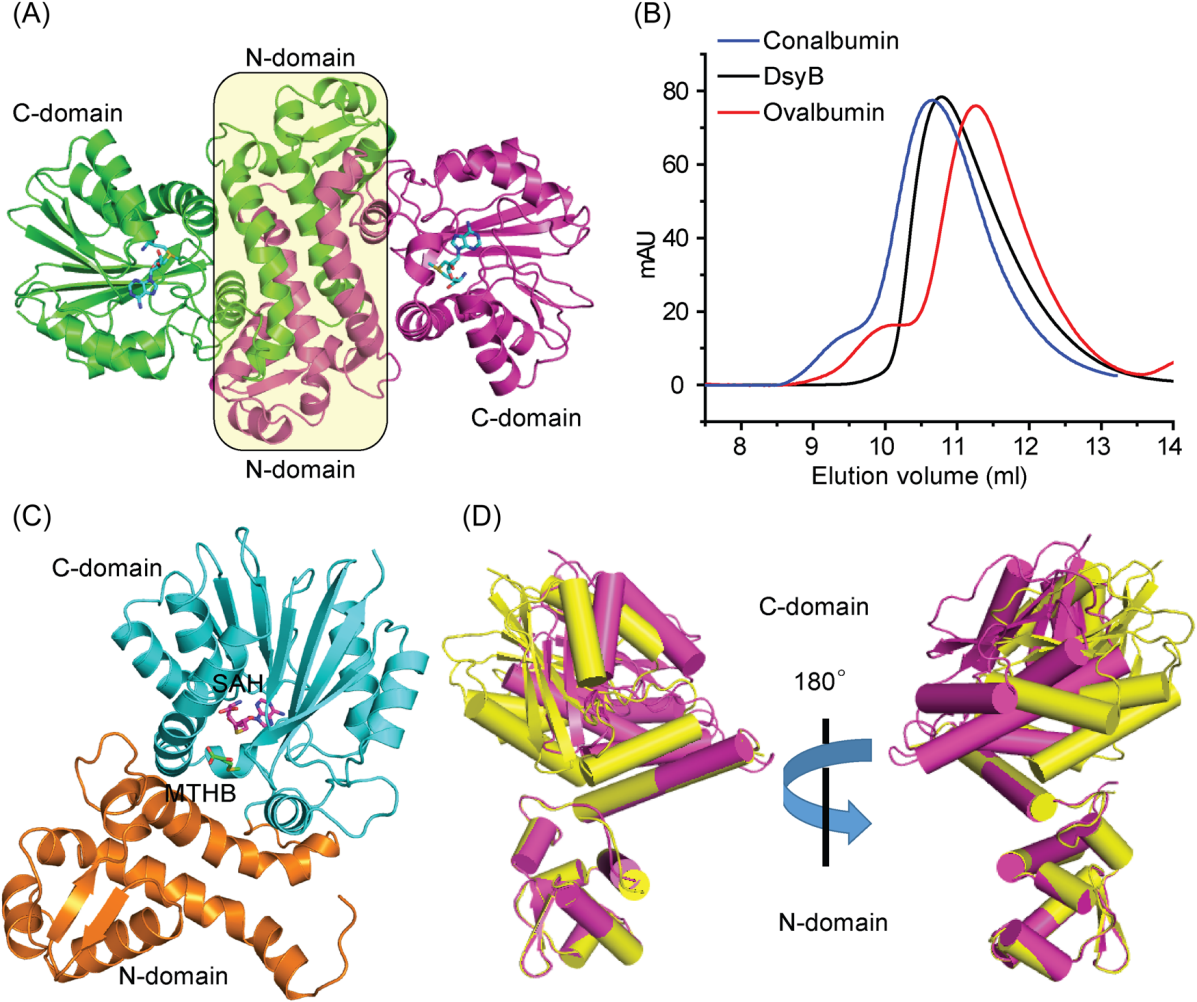
Overall structure analysis of DsyB. (A) Ribbon representation of DsyB dimer. Each DsyB monomer contains an N‐domain and a C‐domain. SAM molecules are shown as sticks colored in cyan. (B) Analysis of the form of DsyB in solution by gel filtration. Conalbumin (molecular mass = 75,000 Da; GE Healthcare) and ovalbumin (molecular mass = 43,000 Da; GE Healthcare) were used as markers. The predicted molecular mass of DsyB monomer is 37,215 Da. (C) The overall structure of DsyB‐SAH‐MTHB complex. The SAH molecule (colored in purple) and the MTHB molecule (colored in green) are represented as sticks. (D) Superimposed structures of DsyB with (colored in purple) and without (colored in yellow) binding the SAM molecule.

Crystals of the DsyB‐SAM complex belonged to the *P*2_1_2_1_2_1_ space group, with four molecules arranged as a tetramer in the asymmetric unit. Each DsyB molecule contains two domains, an N‐terminal domain (N‐domain, Met1‐Ala125) and a C‐terminal domain (C‐domain, Thr126‐Glu337), which can be seen binding to the SAM molecule (Figure 4A). The DsyB C‐domain contains seven β‐strands surrounded by six α‐helices, which together adopt the typical Rossmann‐like α/β fold of Class I SAM‐MTs (Figure 4A). Structural analysis showed that two DsyB monomers are tightly intertwined, mainly through interactions of residues from the N‐domains of two adjacent monomers (Figure 4A). Analysis of DsyB using the PISA server (http://www.ebi.ac.uk/msd‐srv/prot_int/pistart.html) predicted the DsyB dimer to be stable in solution. Indeed, gel filtration analysis indicated that DsyB is likely a dimer in solution (Figure 4B), consistent with the nondenaturing mass spectrometry data above (Figures S2 and S3A). These results indicate that DsyB functions as a dimer in the same way as other SAM‐MTs, whose N‐domains are also responsible for dimerization^19^, ^27^, ^28^. ICP‐MS and LC‐MS analyses showed that the as‐isolated DsyB contained variable metals. However, in the crystal structure of DsyB‐SAM complex, no explicit electron density associated with metals was observed, suggesting that the binding site of metals may be not specific in DsyB.

The crystals of the DsyB‐SAH‐MTHB complex belong to the *P*2_1_ space group (Table S1) and the resulting structure has a similar overall structure to that of the DsyB‐SAM complex (Figure 4C). Interestingly, in this case the MTHB cosubstrate molecule is located between the C‐domain and the N‐domain of one DsyB monomer (Figure 4C).

### The conformational change of DsyB in binding SAM

During the structural refinement of the DsyB‐SAM complex, we found that three monomers (Chain A, B, and C) of the asymmetric unit contained SAM molecules. The structures of these three monomers are similar, with root mean square deviations (RMSDs) of ∼0.5 Å between any two monomers. The Chain D of the DsyB‐SAM complex is not bound to a SAM molecule. The conformation of Chain D is different to the other monomers bound to SAM, with a RMSD of ∼2.4 Å between Chain D and Chain A. Moreover, residues Asp123 to Tyr143 in Chain D exhibited weak electron density, suggesting that this region is highly flexible. By superposing molecules of Chain A and Chain D, we observed that the N‐domains of Chain A and Chain D are almost completely aligned, whereas the C‐domain rotates ∼10° as a rigid body (Figure 4D). These structural differences indicate that DsyB possesses two conformations: an “open” form and a “closed” form. Although DsyB can bind SAM and MTHB individually (Figure S3B), the binding of SAM triggers the conformational change of DsyB from the “open” form to the “closed” form, shrinking the cavity between the N‐domain and the C‐domain of DsyB and possibly promoting the subsequent binding of MTHB.

The structure of the DsyB‐SAM complex is similar to that of the *Streptosporangium sibiricum* SibL protein (PDB code: 4U1Q), a *C*‐directed Class I SAM‐MT, with an RMSD of ∼1.3 Å between these two structures. SAM binding also triggers the conformational change of SibL from an “open” form to a “closed” form to complete the formation of a binding site for its methyl acceptor 3‐hydroxykynurenine^27^. Similar conformational changes have also been observed in other *C/O*‐directed SAM‐MTs, despite their low sequence identities^19^, ^28^.

### Binding sites of SAM and MTHB

**Figure 5:**
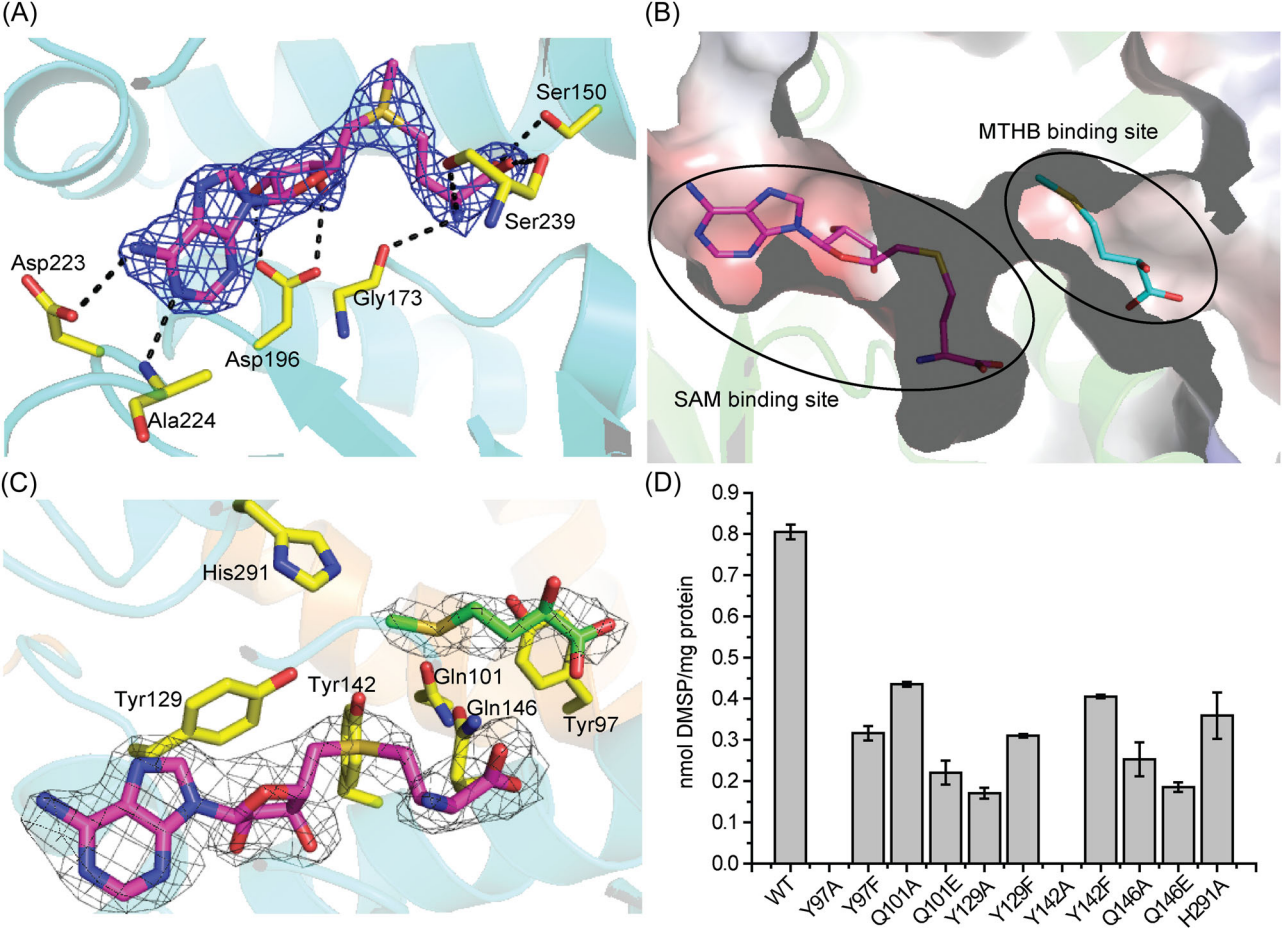
Analyses of residues of DsyB involved in binding SAM and MTHB. (A) Interactions between DsyB residues and SAM. SAM is colored in purple. The possible hydrogen bonds are represented by dashed lines. The 2*F*_*o*_–*F*_*c*_ density for SAM is contoured in blue at 1.5σ. (B) Electrostatic surface of the crystal structure of DsyB. The SAM binding site and the MTHB binding site can be clearly identified. (C) The binding site of MTHB. Residues of DsyB that may participate in binding MTHB are shown in yellow. The 2*F*_*o*_–*F*_*c*_ density for SAH (colored in purple) is contoured in grey at 1.5σ. The 2*F*_*o*_–*F*_*c*_ density for MTHB (colored in green) is contoured in grey at 1.0σ. (D) Enzymatic activities of WT DsyB and site‐directed mutants. The error bar represents the standard deviation of triplicate experiments. WT, wild‐type.

The SAM molecule within the DsyB‐SAM complex (Figure 4A) is bound mainly by hydrogen bonds with residues in the DsyB C‐terminal domain. DsyB residues Asp223 and Ala224 participate in binding the adenine ring of SAM; Asp196 forms hydrogen bonds with the ribose moiety of SAM; and Ser150, Gly173, and Ser239 interacts with the terminal amino acid moiety of SAM (Figure 5A). A similar binding mode was observed between the same DsyB C‐terminal residues for SAH in the DsyB‐SAH‐MTHB complex.

In the DsyB‐SAH‐MTHB complex, the electron density of the MTHB molecule is relatively poor and the distance between SAH and MTHB is more than 6 Å, which is too far to enable the methyl transfer reaction. We speculate that the position of MTHB observed in the structure is not the exact location of MTHB when the reaction occurs under physiological conditions, and that the observed structure represents a state where the MTHB molecule has not completely entered into the active site. Nevertheless, the location of MTHB clearly implies its initial binding site (Figure 5B), and several residues likely involved in the binding of MTHB were identified, including Tyr97 and Gln101 from the N‐domain and Tyr129, Tyr142, Gln146, and His291 from the C‐domain of DsyB (Figure 5C).

To determine the importance of the Tyr97, Gln101, Tyr129, Tyr142, Gln146, and His291 in DsyB binding to MTHB, we performed site‐directed substitutions of these residues and assayed the *in vivo* MTHB *S*‐methyltransferase activity of the resultant variant DsyB derivatives in *R. leguminosarum*. *In vivo* assays were performed in *Rhizobium* because of the sensitivity of *in vitro* assays, see above. Site‐directed mutations of Tyr97, Gln101, Tyr129, Tyr142, Gln146 or His291 severely decreased the enzymatic activity of DsyB (Figure 5D), indicating the potentially important roles of these residues in binding MTHB. In particular, the activity of the Tyr97Ala and Tyr142Ala variants was completely abolished (Figure 5D). However, Tyr97Phe and Tyr142Phe variants maintained >30% residual activity (Figure 5D), suggesting that the elimination of activity of Tyr97Ala and Tyr142Ala substitutions may be caused by the replacement of the aromatic side chain. This mutational analysis suggests that residues likely involved in the binding of MTHB are not essential for catalysis.

Three distinct catalytic mechanisms have been reported for SAM‐MTs, including the proximity and desolvation mechanism, the general acid/base‐mediated mechanism, and the metal‐dependent mechanism^19^. Structural and biochemical analyses indicate that the activity of DsyB is neither metal‐dependent nor catalytic residue‐dependent, but is likely driven by the proximity effect. The DsyB enzyme likely enables favorable orientations of MTHB and SAM molecules that bring the sulfur atom of MTHB in close proximity to the methyl group of SAM.

### The catalytic mechanism of DsyB

**Figure 6:**
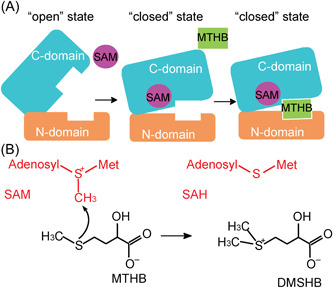
A proposed catalytic mechanism of DsyB. (A) The schematic diagrams of the DsyB conformational change triggered by the binding of SAM. (B) The sulfur atom of MTHB attacks on the methyl group of SAM to generate DMSHB and SAH. The MTHB molecule and DMSHB molecule are shown in black. And the SAM molecule and the SAH molecule are shown in red.

Based on our structural and biochemical results, we propose DsyB first binds a SAM molecule to generate a conformational change from “open” to “closed” state, which may promote the binding of MTHB (Figure 6A). When an MTHB molecule enters the active site, DsyB brings the sulfur atom of MTHB close enough to the methyl group of SAM to allow nucleophilic attack on the methyl group of SAM (Figure 6B). Subsequently, the generated DMSHB and SAH are released, and DsyB can rebind a SAM molecule from the intracellular environment in preparation for the next reaction.

Ecm18, another bacterial *S*‐directed SAM‐MT, converts disulfide in triostin A to the thioacetal linkage in the peptide antibiotic echinomycin through two stages, the methylation of one sulfur atom of the disulfide and the rearrangement of the methylated disulfide to form the thioacetal^29^. Thus, as we predict for DsyB, Ecm18 also uses the PD mechanism for its methyl transfer reaction^29^. Thiopurine *S*‐methyltransferase (TPMT) is a murine *S*‐directed SAM‐MT that methylates 6‐mercaptopurine^30^. Unlike DsyB, TPMT does not contain an N‐terminal domain likely involved in dimerization, as its N‐terminus only constitutes 40 residues^30^. Although Arg147 and Arg221 are possible participants in 6‐mercaptopurine deprotonation, the modest decrease in the enzymatic activities of the corresponding mutants suggests that TPMT may possess the PD strategy for catalysis^30^. *Catharanthus roseus* CrSMT1 is another *S*‐directed SAM‐MT that methylates a broad range of substrates including benzene thiol and furfuryl thiol^31^. Homology modeling suggests that CrSMT1 contains an N‐domain for dimerization^31^, which is similar to DsyB. However, CrSMT1 is thought to use a histidine residue as a general base to deprotonate the thiol group of the substrate^31^. Thus, although the *S*‐directed SAM‐MTs only constitute a small portion of the reported SAM‐MTs^19^, their domain structures and catalytic mechanisms appear diverse.

### Universality of the catalytic mechanism of DsyB

The majority of bacteria containing DsyB are *Rhodobacterales*, which are abundant in marine environments, but this enzyme is also found in some *Rhizobiales* and *Rhodospirillales* (including *N. dentrificans*)^10^, ^32^. To investigate the ubiquity of the DsyB catalytic mechanism, we performed sequence alignment of DsyB proteins from different *Rhodobacterales*, *Rhizobiales*, and *Rhodospirillales* bacteria (Figure S4). Most residues involved in initial MTHB binding (Tyr97, Gln101, Tyr129, Tyr142, Gln146 and His291) and SAM binding (Ser150, Gly173, Asp196, Asp223, Ala224 and Ser239) are highly conserved in DsyB proteins from these marine bacteria, indicating that mechanistic insight gained here for *N. denitrificans* DsyB has universal significance in bacteria containing DsyB.

Eukaryotic DSYB, which may originate from bacterial DsyB, is a key enzyme for DMSP synthesis in many phytoplankton, such as marine haptophytes, dinoflagellates, and some diatoms^12^. DSYB shares ∼33% sequence identity to DsyB, and we predicted the structure of DSYB from *Chrysochromulina tobin* CCMP291 by homologous modeling using SWISS‐MODEL (https://swissmodel.expasy.org/)^33^. Structural alignment of DSYB and DsyB indicated that residues involved in binding MTHB are perfectly superposed (Figure S5). Moreover, sequence alignment of DsyB and DSYB from different marine algae showed that residues which play important roles in DsyB are highly conserved in different DSYB proteins (Figure S6), suggesting that DSYB proteins adopt a similar catalytic mechanism to DsyB.

### DsyB/DSYB are the most abundant and transcribed *S*‐methyltransferase enzymes of known DMSP synthesis pathways in marine microorganisms and environments

Having the identity of the key *S*‐methyltransferases in diverse DMSP synthesis pathways (DsyB/DSYB and TpMMT in the bacterial and algal transamination pathway, and MmtN and BurB in bacterial methylation pathways; Figure 1), we carefully analyzed their presence in marine microorganisms and their abundance and transcript levels in published global metagenome and metatranscriptome datasets to quantify the potential environmental importance of these pathways.

Of the known DMSP synthesis enzymes, DsyB is by far the most abundant in sequenced and/or isolated bacteria (65.8% of cultured DMSP‐producing isolates; Table S2)^7^, ^10^, ^11^, ^34^, ^35^. DsyB is mostly found in alphaproteobacterial *Rhodobacterales*, *Rhizobiales*, and *Rhodospirillales*, but is also sporadically found in, for example, an actinobacterial *Ponticoccus*isolate^7^, and in some *Betaproteobacteria* and *Bacteroidetes* metagenome‐assembled genomes^36^. MmtN has much fewer (14.4% of cultured DMSP‐producing isolates), but equally diverse, host bacteria, being found in *Alphaproteobacteria*, *Gammaproteobacteria*, and *Actinobacteria* (Table S2). Finally, BurB is confined to very closely related *Burkholderia* spp. that likely uses DMSP as an intermediate in toxin production^34^.

**Figure 7:**
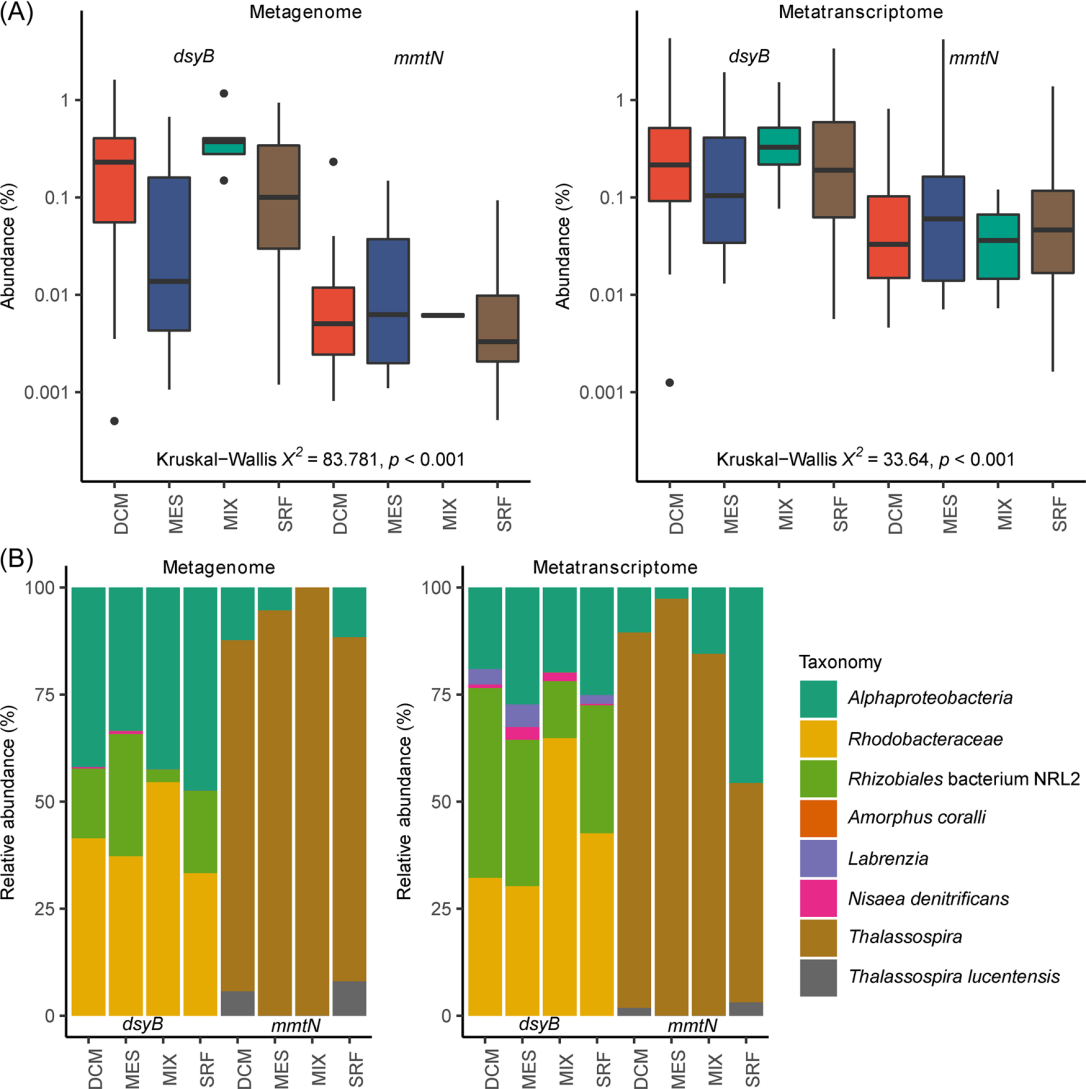
Analysis of MATOU for *dsyB *and *mmtN*. (A) Normalized abundance of *dsyB* and *mmtN* in *Tara* metagenomes and metatranscriptomes, by sampling depth. Abundances are normalized as a percentage of the median gene or transcript abundance of 10 single‐copy marker genes. Box plots show median values (central black line), and lower and upper hinges correspond to the first and third quartiles of the data. Kruskal–Wallis *χ* ^2^ values for comparisons of median abundances between *dsyB* and *mmtN* (across all depths combined) are shown. (B) Taxonomic assignment and relative abundance (as a percentage) of *dsyB* and *mmtN* sequences in the *Tara* metagenomes and metatranscriptomes. Taxa designated *Alphaproteobacteria* lack further taxonomic resolution. DCM, deep chlorophyll maximum layer; MATOU, Marine Atlas of Tara Ocean Unigenes; MES, mesopelagic layer; MIX, epipelagic wind mixed layer; SRF, surface water layer.

This hierarchy of DMSP synthesis gene abundance in bacteria (DsyB > MmtN >BurB) was mirrored in marine environmental data. In the *Tara* Oceans prokaryotic database, both *dsyB* and *mmtN* were found throughout the water column (Figure 7A), but no close homologs of *BurB* (e‐value: <1e−40) were detected. This is consistent with BurB‐mediated DMSP production in *Burkholderia* spp., possibly for toxin production, not being an important process in marine systems. *DsyB* was significantly more abundant than *mmtN* in both the metagenomic (median abundance 0.141% vs. 0.00376%) (Kruskal–Wallis *χ* ^2^ = 83.781, *p* < 0.001) and metatranscriptomic (median abundance 0.2% vs. 0.0364%) (Kruskal–Wallis *χ* ^2^ = 33.64, *p* < 0.001) *Tara* Oceans datasets (Figure 7A). Additionally, *dsyB* was found at 172 and 153 sampling sites (treating each depth as a separate site) in the metagenomes and metatranscriptomes, respectively, whereas *mmtN* was found at only 74 and 63 sites, respectively. Given this, our analysis of median abundance overestimates the contribution of *mmtN* to DMSP production in the global ocean. We, therefore, determined the relative abundance of *dsyB:mmtN* across depths at each sampling site in both metagenomes and metatranscriptomes (Figure S7). *DsyB* was more abundant at almost all sites in the metagenomes, and was more highly expressed across most, though there were a number of locations in the South Atlantic and South Pacific where *mmtN* was dominant (Figure S7). Taxonomic examination of both *dsyB* and *mmtN* sequences in the *Tara* database (Figure 7B) showed that both genes were exclusively from *Alphaproteobacteria*, primarily within the Orders *Rhodobacterales* and *Rhizobiales* for *dsyB*, and the genus *Thalassospira* for *mmtN* (Figure 7B). These data highlight DsyB as the most abundant, transcribed, and likely, important of the known bacterial DMSP synthesis enzymes in marine waters, which likely plays a significant role in the global production of DMSP.

Moving to eukaryotic DMSP synthesis, we carefully analyzed available transcriptome data from marine eukaryotes in the Marine Microbial Eukaryote Transcriptome Sequencing Project (MMETSP)^37^. The TpMMT MTHB *S*‐methyltransferase has only been characterized in the centric diatom *T. pseudonana* CCMP1335, and close homologs (∼70% protein identity) with the same singular domain structure only exist in 17/82 diatom transcriptomes (seven of which also contain DSYB), and no other phytoplankton (Tables S3 and S4)^38^, ^39^, ^40^, ^41^, ^42^, ^43^, ^44^, ^45^, ^46^, ^47^, ^48^, ^49^, ^50^, ^51^, ^52^, ^53^. The next most homologous TpMMT‐like proteins, present in for example, *Thalassiosira oceanica* (EJK59074) and *Fistulifera solaris* (GAX25165) that are more diverse (the methyltransferase domain being <50% identical to TpMMT), contain extra protein domains and, thus, are much larger proteins whose function is unknown. These TpMMT‐like proteins cannot be considered as functional MTHB *S*‐methyltransferase enzymes and are omitted from this study. In contrast, DSYB is found in the transcriptomes of 47/61 dinoflagellates and 24/30 haptophytes, organisms known to produce the highest levels of DMSP per cell (>50 mM)^39^, ^46^. Furthermore, 15/82 diatom transcriptomes, typically known to produce lower cellular DMSP levels (generally <50 mM)^39^, and some *Ochrophyta*, *Cnidaria*, and *Cilophora* transcriptomes also contained DSYB. These data show DSYB to be the most abundant and widespread DMSP synthesis enzyme known in eukaryotic DMSP‐producing organisms.

Within the eukaryotic Marine Atlas of Tara Ocean Unigenes (MATOU), we found both *DSYB* and *TpMMT* within epipelagic (surface water layer [SRF] and deep chlorophyll maximum [DCM]) waters. Initial examination showed *DSYB* to be more abundant in ≤3 µm fractions than in larger fractions (Figure S8). Data from these smaller ≤3 µm fractions that likely contain picoeukaryotes (CoP) were considered together. Likewise, fractions with a minimum filter size of ≥3 µm that likely exclude picoeukaryotes (ExP) were also considered together. Abundance was not significantly different between SRF and DCM sampling depths for either CoP or ExP *DSYB* (Kruskal–Wallis *χ* ^2^ = 0.113, *p* = 0.74, and Kruskal–Wallis *χ* ^2^ = 0.004, *p* = 0.95, respectively), or for CoP or ExP *TpMMT* (Kruskal–Wallis *χ* ^2^ = 0.102, *p* = 0.75, and Kruskal–Wallis *χ* ^2^ = 0.194, *p* = 0.66, respectively), and as such these sampling depths were combined for the purposes of comparative analyses between *DSYB* and *TpMMT* abundance.

**Figure 8:**
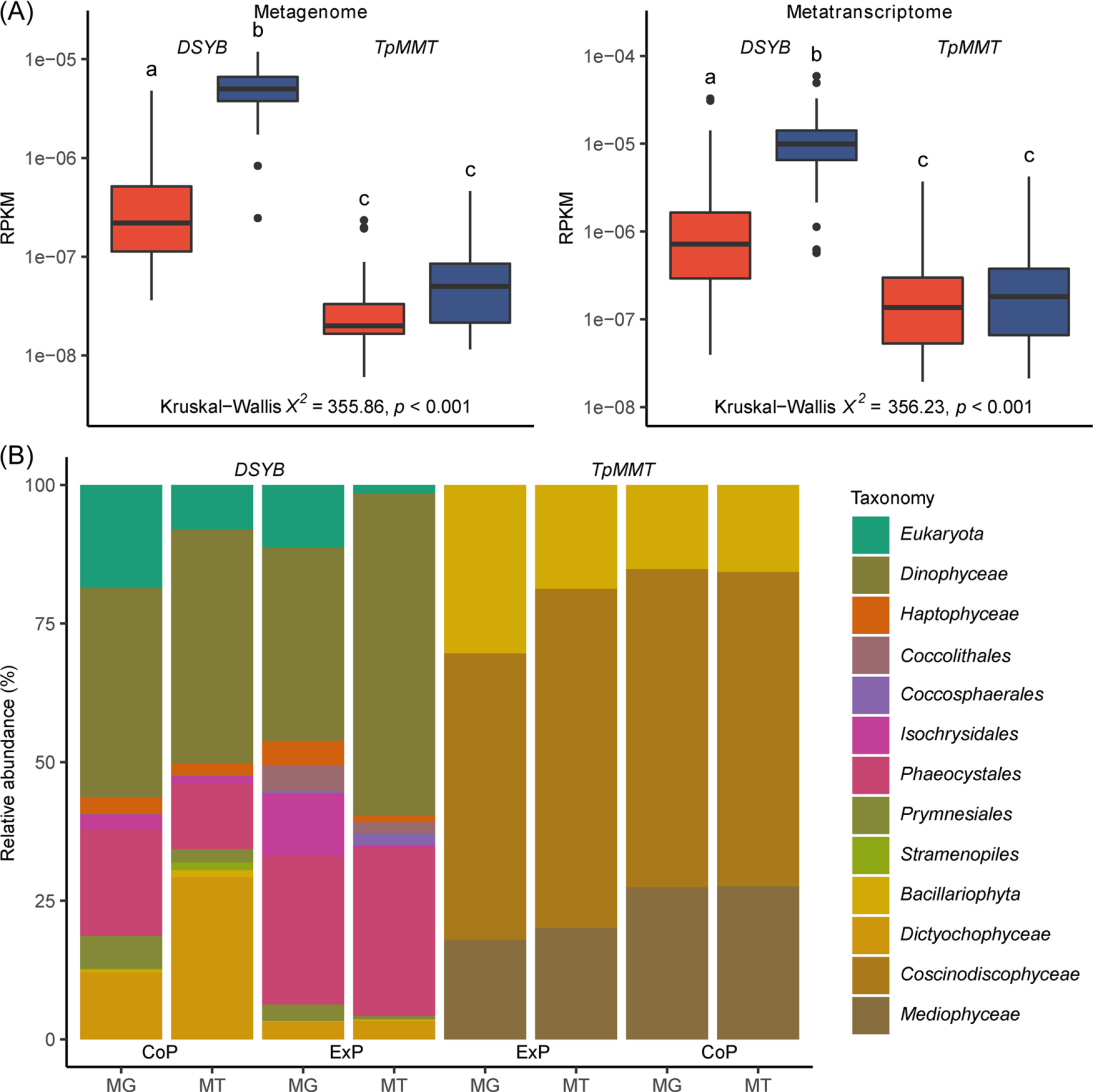
Analysis of MATOU for *DSYB *and *TpMMT*. (A) Normalized abundance of *DSYB* and *TpMMT* in MATOU metagenome and metatranscriptomes, by fractions containing picoeukaryotes (CoP) (i.e., with a minimum filter size of <3 µm) (blue) and fractions excluding eukaryotes (ExP) (i.e., with a minimum filter size of ≥3 µm) (red). DCM and SRF depths are combined for the purposes of this analysis. Abundances are normalized as reads per kilobase per million mapped reads (RPKM). Box plots show median values (central black line), and lower and upper hinges correspond to the first and third quartiles of the data. Kruskal–Wallis *χ* ^2^ values for comparisons between *DSYB*/*TpMMT* and fraction abundance are shown. Letters denote gene or transcript/fraction combinations that are significantly different (*p* < 0.05) by post‐hoc Dunn's test, using Holm's correction. (B) Taxonomic assignment and relative abundance (as a percentage) of *DSYB* and *TpMMT* sequences in the MATOU metagenome (MG) and metatranscriptome (MT). Taxonomy is reported as Phylum (if available) for *DSYB* and as Class (if available) for *TpMMT*. Taxa designated *Eukaryota* lack further taxonomic resolution.

Metagenomes derived from the MATOU data set revealed that *DSYB* was significantly more abundant than *TpMMT* in both CoP and ExP fractions (Figure 8A; *DSYB* vs. *TpMMT* CoP median abundance 4.99e−06 vs. 5.01e−08 RPKM, post‐hoc Dunn's test *z* = 16.22, *p* < 0.001, ExP median abundance 2.2e−07 vs. 1.99e−08 RPKM, post‐hoc Dunn's test *z* = 6.97, *p* < 0.001). Similarly, *DYSB* was significantly more abundant than *TpMMT* in MATOU derived metatranscriptomes (Figure 8A; *DSYB* vs. *TpMMT* CoP median abundance 9.89e−06 vs. 1.81e−07 RPKM, post‐hoc Dunn's test *z* = 15.16, *p* < .001, ExP median abundance 7.21e−07 vs. 1.36e−07 RPKM, post‐hoc Dunn's test *z* = 7.33). *DSYB* was also significantly more abundant within the CoP fraction than the ExP fraction in the metagenome (Figure 8A; median abundance 4.99e−06 vs. 2.2e−07, post‐hoc Dunn's test *z* = 11.30, *p* < 0.001), and in the metatranscriptome (Figure 8A; median abundance 9.89e−06 vs. 7.21e−07, post‐hoc Dunn's test *z* = 12.20, *p* < 0.001). In contrast, *TpMMT* abundance was not significantly different between CoP and ExP fractions in either the metagenome (Figure 8A; median abundance 5.01e−08 vs. 1.99e−08, post‐hoc Dunn's test *z* = 1.61, *p* = 0.11) or the metatranscriptome (Figure 8A; median abundance 1.81e−07 vs. 1.36e−07, post‐hoc Dunn's test *z* = 0.92, *p* = 0.35). Again, these analyses likely overestimated the abundance of *DSYB* in the ExP fraction, because, in the metagenome, *DSYB* was detected at 138/140 CoP fraction sites, but was only found at 178/272 ExP fraction sites (*χ* ^2^ [1, *N* = 412] = 56.77, *p* < 0.001). *TpMMT* abundance was also likely overestimated as *TpMMT* was detected at 90/140 CoP fraction sites, and at 39/272 ExP fraction sites (*χ* ^2^ [1, *N* = 412] = 107.21, *p* < 0.001). As such, *DSYB* was detected significantly more frequently in CoP (*χ* ^2^ [1, *N* = 280] = 54.41, *p* < 0.001) and ExP (*χ* ^2^ [1, *N* = 544] = 148.12, *p* < 0.001) fraction sites than *TpMMT*. Similarly, within the metatranscriptome, *DSYB* was detected at 139/140 CoP fraction sites and at 251/272 ExP fraction sites (*χ* ^2^ [1, *N* = 412] = 898, *p* < 0.01). *TpMMT* was detected at 94/140 CoP fraction sites, and at 99/272 ExP fraction sites (*χ* ^2^ [1, *N* = 412] = 35.09, *p* < 0.001). Again, *DSYB* was detected at significantly more CoP (*χ* ^2^ [1, *N* = 280] = 51.78, *p* < 0.001) and ExP (*χ* ^2^ [1, *N* = 544] = 185.10, *p* < 0.001) fraction sites than *TpMMT* in the metatranscriptome data. Given the greater abundance of *DSYB* over *TpMMT* in the environmental data, and that the majority of environmental *DSYB* sequences are likely from dinoflagellates and/or haptophytes, known to be high producers^39^, ^46^,  of DMSP compared to *TpMMT* in the generally low‐producing diatoms^39^, DSYB is currently the most important known DMSP synthesis enzyme (Figure 8B).

To conclude, DMSP is an abundant and ecologically important organosulfur compound. DsyB/DSYB enzymes catalyze the committed *S*‐methylation of MTHB to generate DMSHB, which is the key step of the transamination pathway for DMSP synthesis in most bacteria and algae^10^, ^11^, ^12^. Furthermore, DsyB/DSYB enzymes are present in the most prodigious DMSP‐producing haptophyte and dinoflagellate phytoplankton, and represent the most abundant and transcribed *S*‐methylase genes of known DMSP synthesis pathways in marine waters. In this study, we solved the first crystal structures of bacterial DsyB‐SAM and DsyB‐SAH‐MTHB complexes and demonstrated the conversion of SAM and MTHB into SAH and DMSHB. Based on structural and mutational analyses, the catalytic mechanism of DsyB is proposed, and has universal significance in bacteria containing DsyB, and in marine algae containing DSYB. Our results provide novel insights into DMSP synthesis, shedding light on the global sulfur cycling.

## MATERIALS AND METHODS

### Bacterial strains and growth conditions

*E. coli* BL21 (DE3) was grown in lysogeny broth (LB) medium at 37°C. *R. leguminosarum* J391 was grown in TY^54^ complete medium or Y^54^ minimal medium (10 mM NH_4_Cl as nitrogen source) at 28°C. *L. aggregata* J571 (*dsyB* ^−^) was grown in YTSS^55^ complete medium or marine basal minimal (MBM)^56^ medium (0.5 mM NH_4_Cl as nitrogen source) at 30°C. *N. denitrificans* DR41_21 (DSM 18348), purchased from DSMZ, Germany, was cultured in the Difco 2216 medium (http://www.dsmz.de/) or MBM medium at 30°C under different salinity and nitrogen levels for differential DMSP production experiments. Standard conditions were 10 mM NH_4_Cl and 35 practical salinity units (PSU) compared to 5 PSU (low salinity) or 50 PSU (high salinity). Cultures were sampled for DMSP and reverse transcription‐quantitative polymerase chain reaction (RT‐qPCR) work in exponential‐phase growth (after ∼7 days). For nitrogen starvation experiments, exponential phase cells grown under standard conditions were harvested and incubated overnight in standard MBM media with no added NH_4_Cl. Where necessary, 10 mM succinate was used as a carbon source and antibiotics were added at the following concentrations: gentamicin (20 µg ml^−1^), streptomycin (400 µg ml^−1^), kanamycin (20 µg ml^−1^), ampicillin (100 µg ml^−1^), spectinomycin (200 µg ml^−1^).

### General *in vivo* and *in vitro* genetic manipulations

Plasmids (Table S5) were transferred to *E. coli* by transformation, and *R. leguminosarum* J391 or *L. aggregata* J571 by conjugation using the helper plasmid pRK2013^57^. Routine restriction digestions and ligations for cloning were performed essentially as in ref.^58^. The oligonucleotide primers used in this study were synthesized by Eurofins Genomics (Table S6). Sequencing of plasmids and PCR products was performed by Eurofins Genomics.

The *dsyB* gene was amplified from *N. denitrificans* genomic DNA and cloned into the pET22b (Novagen) for the expression of DsyB with a C‐terminal His‐tag or into pLMB509 for expression in *Rhizobium* and *Labrenzia* ^59^. Amino acid substitution mutations in DsyB were generated using QuickChange^®^ mutagenesis kit (Agilent Technologies) and the primers in Table S6. All site‐directed mutant (SDM) variant plasmids were verified by DNA sequencing.

### Reverse transcription‐quantitative polymerase chain reaction

RNA was isolated from 100 ml *N. denitrificans* cultures using RNeasy Mini Kit (Qiagen) according to the manufacturer's protocol with some modifications. On‐Column DNase digestion was performed with RNase‐Free DNase Set (Qiagen). Reverse transcription of 1 µg of DNA‐free RNA per sample was done using the QuantiTect Reverse Transcription Kit (Qiagen). PCR on RNA and complementary DNA (cDNA) samples confirmed that RNA samples were DNA‐free.

Primers for RT‐qPCR for *N. dentrificans dsyB* and housekeeping genes *recA* and *gyrB*, were designed using Primer5^60^ (Table S6). The qPCR experiments were performed on a StepOnePlus instrument (Applied Biosystems). Quantification was performed using the SYBR® Green JumpStart™ Taq ReadyMix™ (Sigma‐Aldrich) following the manufacturer's instructions. Reactions (20 µl) contained 2 µl cDNA and 0.8 µl primers (10 µM), with an annealing temperature of 55°C. For each condition and gene, the cycle threshold (*C*_t_) values of triplicate technical and biological replicates were averaged. Relative expression levels were determined with efficiency correction^61^. *dsyB* transcription was displayed as normalized fold change to the standard condition.

### DsyB enzyme assays *in vivo*

To measure MTHB *S*‐methyltransferase activity from pLMB509 clones (and SDM derivatives) in *R. leguminosarum* J391, cultures were grown overnight in TY complete medium. Then 1 ml of culture was centrifuged at 20,000*g* for 2 min, resuspended in the same volume of Y medium and then diluted 1:100 into 5 ml Y with 0.5 mM DL‐MTHB (55875; Sigma‐Aldrich), 10 mM taurine (to induce expression; T0625; Sigma‐Aldrich) and gentamicin, and incubated for 60 h at 28°C before sampled for gas chromatography (GC) analysis to determine the amount of DMSP product (see below).

To measure MTHB *S*‐methyltransferase activity from pLMB509 clones expressing the *dsyB* gene in the *L. aggregata dsyB* ^–^ mutant strain J571, cultures were grown overnight in YTSS complete medium. Following incubation, 1 ml of culture was then centrifuged at 20,000*g* for 2 min, resuspended in the same volume of MBM medium and then diluted 1:50 into 5 ml MBM with 10 mM taurine (to induce expression; Sigma‐Aldrich), gentamicin and rifampicin, and incubated for 24 h at 30°C. DL‐MTHB (0.5 mM) was added as substrate to the cultures and these were incubated for 4 h at 30°C before sampled for GC and protein estimation by the Bradford assay.

To measure DMSHB/DMSP in *Rhizobium* or *Labrenzia* assay mixtures, 200 µl of culture was added to a 2 ml glass serum vial, then 100 µl 10 M NaOH was added and vials were crimped with polytetrafluoroethylene/rubber crimp caps immediately. Vials were incubated at 80°C for 10 min (to capture DMSHB as well as DMSP) and then for 24 h at room temperature in the dark before being monitored by GC assay. All GC assays involved measurement of headspace DMS using a flame photometric detector (Agilent 7890A GC fitted with a 7693 autosampler) and an HP‐INNOWax 30 m × 0.320 mm capillary column (Agilent Technologies J&W Scientific). Calibration curves were produced by alkaline lysis of DMSP standards in water. The detection limit for headspace DMS from DMSP was 0.015 nmol and from DMSHB was 0.3 nmol. DsyB activity is expressed as nmol DMSHB/DMSP min^−1^ mg protein^−1^. Protein concentrations were determined using the Bradford method (BioRad). Control assays of *Rhizobium* or *Labrenzia* J571 containing pLMB509 were carried out, as above, and gave no detectable DsyB activity.

### Protein expression and purification

*E. coli* BL21 (DE3) containing pET22b::*dsyB* clones were cultured in an LB medium containing ampicillin at 37°C. At mid‐exponential growth (OD_600_ 0.5–0.7), 0.5 mM isopropyl β‐*D*‐1‐thiogalactopyranoside was added and the cells were incubated at 20°C for 16 h. Cells were harvested by centrifugation (20 min, 7500*g*, 4°C), washed, and resuspended in 25 mM Tris‐HCl, pH 8.0, 150 mM NaCl. Cell lysis was performed by three passages through a French Press (16,000 psi), unbroken cells and cell debris were removed by centrifugation (30 min, 5500*g*, 4°C) and the supernatant was recovered and subjected to centrifugation (60 min, 185,000*g*, 4°C) to pellet the membrane fraction. The soluble cell lysate was applied to a slurry of Ni‐NTA resin (Qiagen) at a 3:1 ratio for 90 min with shaking at 4°C. The lysate/slurry mix was loaded into Econo‐Pac polypropylene columns, washed with 50 mM Tris‐HCl, 250 mM NaCl, 20 mM imidazole, pH 8.0, and DsyB was eluted in 5 × 1 ml fractions using 50 mM Tris‐HCl, 250 mM NaCl, 250 mM imidazole, pH 8.0. Fractions containing DsyB were concentrated and buffer exchanged into 2 ml of 50 mM Tris‐HCl, 100 mM NaCl, pH 8.0 and applied to a Superdex 200 10/300GL preparative grade gel filtration column (Cytiva). The purified protein was flash‐frozen in liquid nitrogen and stored at −80°C until required.

### DsyB enzyme assays *in vitro*

*L. aggregata dsyB* ^−^ J571^10^ was grown to late exponential phase in MBM. Cell lysates were prepared by centrifuging 100 ml of culture for 10 min at 2500*g*. The pellet was washed and resuspended with 20 ml 20 mM 4‐(2‐hydroxyethyl)‐1‐piperazineethanesulfonic acid (HEPES), 150 mM NaCl, pH 7.5 before cell lysis via French press (16,000 psi). The cell lysate was heat‐treated at 80°C for 10 min to denature proteins, then applied to a 10 ml PD10 column, eluted over 10 ml, and collected in 1 ml fractions. DsyB activity was monitored by performing *in vitro* enzyme assays with 50 µl of the separate heat‐killed extract fractions, 1 mM SAM (Sigma‐Aldrich), 1 mM DL‐MTHB and 1.97 µM DsyB or no protein (control) in 400 µl total volume. Experiments were done as above with purified DsyB without addition of heat‐killed extracts, but these gave no activity (data not shown). Reactions were incubated for 30 min at 28°C and then 800 µl of activated charcoal (38 mg ml^−1^ in 0.1 M acetic acid) was added to the samples and mixed. Samples were centrifuged at 14,000*g* for 15 min and the supernatant was retained. For GC analysis, 200 µl of the supernatant was added to a 2 ml vials with 10 M NaOH (100 µl) and was immediately crimped. Crimped vial was then heated to 80°C for 10 min and incubated in the dark at 22°C for 16 h. These samples were subsequently used for DMS quantification by GC analysis (as above). No DMS was produced from the no DsyB protein controls.

For kinetics analysis of *N. denitrificans* DsyB, the as‐isolated protein was activated by addition of 400 µl heat‐killed cell lysate fractions liberated from the *L. aggregata dsyB^–^* deletion mutant. *K*_m_ and *V*_max_ values were determined by nonlinear analysis using 1.97 µM DsyB and 0–2 mM SAM (fixed at 1 mM for DL‐MTHB kinetic work), or 0–2 mM DL‐MTHB (fixed at 1 mM for SAM kinetics work; Figure 2). The reaction mixture was incubated at 28°C for 30 min before detection of DMSHB as above. Origin version 8.5 was used to calculate *K*_m_.

### Mass spectrometry analysis

LC‐MS was used to confirm the mass of intact (but denatured) DsyB, and also for the analysis of small molecules. For analysis of DsyB, denaturing LC‐MS was conducted using a Bruker microQTof‐QIII electrospray ionization time of flight mass spectrometer, operating in positive mode. The spectrometer was calibrated with ESI‐L Low Concentration Tuning Mix (Agilent Technologies). Samples were prepared by 10‐fold dilution of ∼100 µM DsyB protein solution with 2% (vol/vol) acetonitrile and 0.1% (vol/vol) formic acid to 0.5 ml. Samples were chromatographically separated by an UltiMate 3000 HPLC system (Dionex) fitted with a ProSwift reversed phase RP‐1S column (4.6 × 50 mm; Dionex). Hystar (Bruker Daltonics) was used to coordinate mass spectrometer and high‐performance liquid chromatography (HPLC) operations. Bound proteins were eluted using an isocratic gradient (2%–100% B) at a flow rate of 0.2 ml min^−1^ using the following solvents: Solvent A (water, 0.1% [vol/vol] formic acid); and Solvent B (acetonitrile, 0.1% [vol/vol] formic acid). The eluant was continuously infused into the source of the mass spectrometer operating with the following parameters: dry gas flow 8 l min^−1^; nebulizer gas pressure 1.8 bar; dry gas 240°C; capillary voltage 4500 V; offset 500 V; collision RF 650 Vpp.

Mass spectrometric analysis of small molecule substrates and products in DsyB assay mixtures was performed using HILIC^62^, which is particularly useful for the separation of small polar compounds such as MTHB or DMSHB. HILIC‐MS^63^ experiments were performed using the same mass spectrometer and HPLC system as above, but with the latter fitted with a Luna NH_2_ column (2 × 100 mm) (Phenomenex). For HILIC chromatography, the following solvents were freshly prepared: Solvent A (95% [vol/vol] aqueous 5 mM ammonium formate, pH 3.75, 5% [vol/vol] acetonitrile); Solvent B (95% [vol/vol] acetonitrile, 5% [vol/vol] aqueous 100 mM ammonium formate, pH 3.75). Standard compounds (SAM, DL‐MTHB, SAH [Sigma‐Aldrich], DMSHB)^10^ were used to calibrate the elution profile of the HILIC column. Samples were brought to 92% (vol/vol) acetonitrile and loaded onto a column pre‐equilibrated with Solvent B. An optimized HILIC gradient was applied and compounds were eluted (0.6 ml min^−1^) using the HILIC gradient between Solvent A and Solvent B, as previously described^10^. The eluant was continuously infused into the source of the mass spectrometer (optimized for 50–600 *m/z*) with the following parameters: dry gas flow 8.5 l min^‐1^; dry gas 200°C; nebulizer pressure 1.2 bar; capillary voltage 4500 V; offset 500 V; collision RF 400 Vpp. Each HILIC‐MS run contained an internal sodium formate calibration segment at the end of the run.

Nondenaturing MS (often referred to as native MS), in which noncovalently bound protein–cofactor, simple protein–protein, or even multiprotein interactions are preserved^64^, ^65^, was used to investigate substrate binding and confirm the presence of dimeric DsyB. Before analysis, protein samples were exchanged into 250 mM ammonium acetate, pH 8.0, using Zeba spin (Thermo Scientific) or PD mini trap (Cytiva) desalting columns and infused (0.3 ml/h) directly into the ESI source of the Bruker microQTof‐QIII mass spectrometer with the following parameters: dry gas flow 4 l min^−1^; nebulizer gas pressure 0.8 bar; dry gas temperature 190°C; capillary voltage 3000 V; capillary offset 500 V; ion energy 8 eV; collisional RF 1500 Vpp; collision cell voltage 5 V; and, ion transmission range 1500–5500 *m/z*.

Processing, isotope pattern simulation and analysis of denaturing LC‐MS, HILIC‐MS, and nondenaturing MS data were carried out using Compass Data Analysis version 4.1. For denaturing LC‐MS and nondenaturing MS, neutral mass spectra were generated using ESI compass Maximum Entropy deconvolution algorithm version 1.3. Proteins masses were reported from peak centroids representing the isotope average neutral mass and compared to predicted masses (Expasy)^66^.

### DsyB activity detection using HILIC‐MS

Heat‐killed J571 fractions that restored MTHB *S*‐methyltransferase were added to pure DsyB as above to yield activated samples for analysis here. Samples of as‐isolated DsyB prepared in HEPES buffer or activated DsyB, were immediately desalted (PD10; Cytiva) into 25 mM Tris, 100 mM NaCl, pH 8.0 before conducting HILIC or nondenaturing MS experiments. The methyltransferase activity of DsyB was measured using DL‐MTHB and SAM as substrates, as previously described^17^, with a slight modification. The reaction mixture (20 μl) consisted of 14 μl water, 2 μl of buffer (100 mM Tris‐HCl, pH 7.5), 0.5 μl of 20 mM DL‐MTHB, 1.5 μl of 32 mM SAM as cosubstrate, and 2 μl of enzyme solution (DsyB in range 7–30 µM depending on particular experiment). The reaction was incubated at 25°C, overnight and quenched by the addition of 230 μl acetonitrile. Samples were analyzed immediately by mass spectrometry.

### Crystallization and data collection

The purified DsyB protein was concentrated to ∼8 mg ml^‐1^ in the buffer containing 100 mM NaCl and 10 mM Tris‐HCl (pH 8.0). Initial crystallization trials for DsyB were performed using the sitting drop vapor diffusion method at 20°C. To obtain crystals of DsyB‐SAM complex, the purified DsyB protein was mixed with 1 mM SAM at 4°C for 30 min. Diffraction‐quality crystals of DsyB‐SAM complex were obtained in hanging drops containing 0.1 M HEPES (pH 7.5), 0.2 M NaCl and 25% (wt/vol) polyethylene glycol (PEG) 3350 after 1‐week incubation at 20°C. Crystals of the DsyB‐SAM complex Se derivative were obtained in hanging drops containing 0.1 M Bis‐Tris propane (pH 7.5), 0.2 M sodium acetate, and 20% (wt/vol) PEG 3350 after 1‐week incubation at 20°C. To obtain crystals of DsyB‐SAH‐MTHB complex, the purified DsyB protein was mixed with SAH (1 mM) and DL‐MTHB (1 mM) at 4°C for 30 min. Crystals of DsyB‐SAH‐MTHB complex were obtained in hanging drops containing 0.1 M Tris (pH 8.0), 0.2 M NaCl, and 20% PEG 4000 after 1‐week incubation at 20°C. X‐ray diffraction data were collected on the BL18U1&BL19U1 beamlines at the Shanghai Synchrotron Radiation Facility. The initial diffraction data sets were processed by the HKL3000 program^67^.

### Structure determination and refinement

The crystals of DsyB‐SAM complex belong to the *P*2_1_2_1_2_1_ space group, while the crystals of DsyB‐SAH‐MTHB complex belong to the *P*2_1_ space group. The structure of DsyB‐SAM complex Se derivative was determined by SAD phasing. The crystal structures of DsyB‐SAM complex and DsyB‐SAH‐MTHB complex were determined by molecular replacement using the CCP4 program Phaser^68^ with the structure of the Se derivative as the search model. The refinements of these structures were performed using Coot^69^ and *Phenix* ^70^. All the structure figures were produced with the PyMOL (http://www.pymol.org/).

### Analyses of DMSP synthesis genes in cultured microorganisms

The presence or absence of DMSP synthesis genes in 111 cultured DMSP‐producing bacteria (published since the discovery of bacterial DMSP synthesis^7^, ^10^, ^11^, ^34^, ^35^), was analyzed (Table S2). This is based on previously published work that analyzed their sequenced genomes and/or used degenerate primers to detect the presence of *burB*, *dsyB*, and/or *mmtN*. Percentage abundances were calculated for all three DMSP synthesis genes within these cultured organisms, as well as those containing both *dsyB* and *mmtN*.

Eukaryotic transcriptomes from the MMETSP^37^ (Tables S3 and S4) were analyzed for the presence of *DSYB* and/or *TpMMT* through tblastn searches against DSYB^12^ and TpMMT^17^ sequences whose enzyme activity had been previously demonstrated. These were manually curated to confirm identity (*e* value cutoff of 1e^−30^ for DSYB), although TpMMT has only been shown to be functional in *T. pseudonana*, we did not assume any sequences below 70% identity to *T. pseudonana* TpMMT to be functional. Strains confirmed to contain *DSYB* and/or *TpMMT* are listed in Table S3 and summarized in Table S4, alongside literature reporting the presence of DMSP synthesis in that particular strain (if tested).

### Metagenome and metatranscriptome analyses

Verified sequences^7^, ^10^, ^11^, ^12^, ^71^, were aligned using ClustalOmega^72^, and profile hidden Markov models (hmms) of *dysB*, *DSYB*, and *mmtN* were constructed using the hmmbuild function of hmmer 3.3^73^. *Tara* metagenome (OM‐RGC_v2_metaG/MATOU_v1_metaG) (prokaryotic/eukaryotic, respectively), and metatranscriptome (OM‐RGC_v2_metaT/MATOU_v1_metaT) sequences together with their abundances and taxonomic assignations were downloaded from the Ocean Gene Atlas site^74^ using an hmmsearch *e*‐value threshold of 1e−70 (*dsyB*), 1e−80 (*DSYB*), or 1e−60 (*mmtN*). A blastp search (*e*‐value threshold of 1e−80) was used for *TpMMT*, using the *T. pseudonana TpMMT* sequence as query. Environmental *dsyB*/*DSYB* sequences were aligned with *N. denitrificans dsyB* using ClustalOmega^72^, and sequences that did not possess all six essential residues were excluded from further analysis. Environmental *TpMMT* sequences greater than 400 amino acids in length were also excluded from further analysis. Prokaryotic sequence abundances were normalized using the median abundance of 10 single‐copy marker genes/transcripts^75^. This gave abundance as a percentage of single‐copy gene abundance (equivalent to the percentage of cells containing a copy) in the metagenome, and transcription as a percentage of single‐copy gene transcription in the metatranscriptome. These marker gene/transcript abundances were downloaded from the Ocean Gene Atlas using the hmm profiles developed by Milanese et al.^75^ with an *e*‐value threshold of 1e−80. The MATOU_v1_metaG (metagenomic) database featured few MIX and MES sampling sites (2 and 7, respectively), limiting the power of comparative analysis between sampling depths, thus, these sites were excluded from the analysis. Statistical analysis was performed in R (version 4.02) using RStudio.

## AUTHOR CONTRIBUTIONS

Jonathan D. Todd and Yu‐Zhong Zhang designed the research. Nick E. Le Brun and Yu‐Zhong Zhang directed the research. Chun‐Yang Li, Jason C. Crack, Simone Newton‐Payne, Andrew R. J. Murphy, Benjamin J. Pinchbeck, and Shun Zhou performed the experiments. Xiu‐Lan Chen, Beth T. Williams, Ming Peng, and Yin Chen helped in the data analysis. Chun‐Yang Li, Jason C. Crack, Beth T. Williams, Andrew R. J. Murphy, and Xiu‐Lan Chen wrote the manuscript. Xiao‐Hua Zhang, Yin Chen, and Nick E. Le Brun edited the manuscript.

## ETHICS STATEMENT

This article does not contain any studies with human participants or animals performed by any of the authors.

## CONFLICT OF INTERESTS

The authors declare no conflict of interests.

## Supporting information

Supporting information.

Supporting information.

Supporting information.

Supporting information.

## Data Availability

The structures of DsyB‐SAM complex and DsyB‐SAH‐MTHB complex have been deposited in the Protein Data Bank under the accession codes 7WDQ and 7WDW, respectively.
