# Supplementary material for: Mechanistic insights into the key marine dimethylsulfoniopropionate synthesis enzyme DsyB/DSYB
Source: mLife. 2022 Jun 15;1(2):114–30. doi: 10.1002/mlf2.12030 (PMC10989797; doi:10.1002/mlf2.12030)
Supplement: Supplementary file 1 — Supporting information. [file MLF2-1-114-s001.docx]

**Mechanistic insights into the key marine dimethylsulfoniopropionate synthesis enzyme DsyB/DSYB**

**Supplementary materials:**


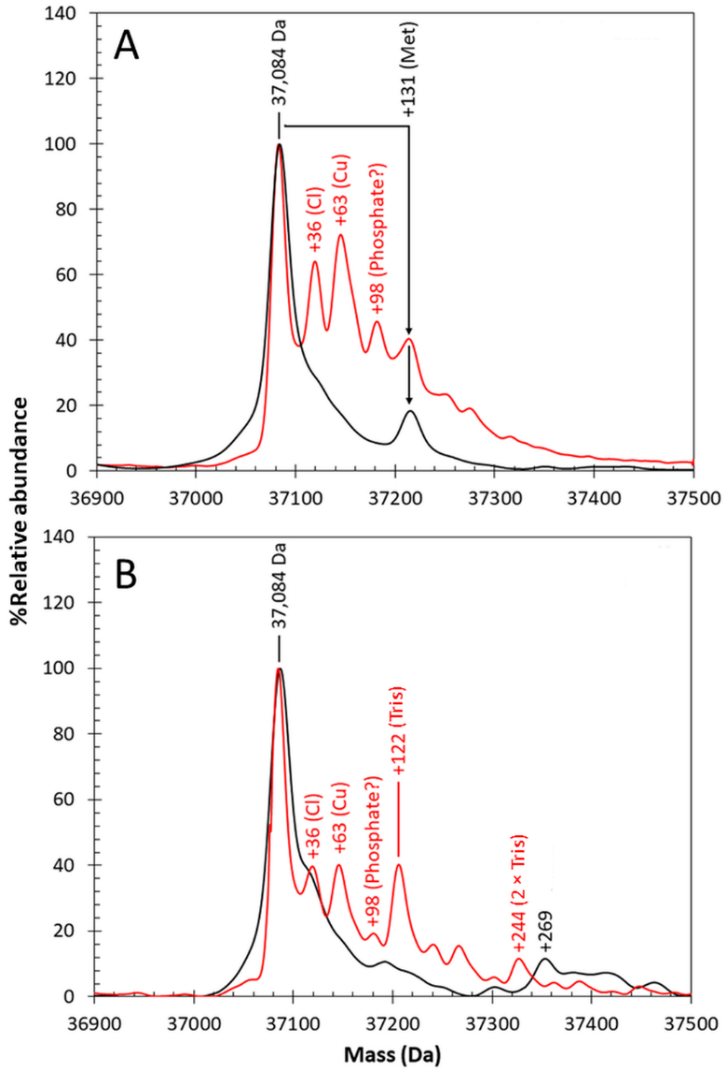


**Fig. S1. Mass spectrometric analyses of DsyB**. (A) Deconvoluted LC-MS spectrum of as-isolated DsyB (~10 μM) in 25 mM Tris, 100 mM NaCl pH 8 (black line), and deconvoluted non-denaturing spectrum of as-isolated DsyB (~20 μM) in 250 mM ammonium acetate pH8.0 buffer (red line). The partial cleavage of the N-terminal Met residue is indicated by the observation of two protein peaks separated by the mass of a single Met residue (131 Da). (B) As in (A) but DsyB was pre-activated by addition of lysate from *L. aggregata* *dsyB* deletion strain. The mass of the main protein peak is indicated and adduct species are labelled with the additional mass and origin (if known). The DsyB sample used for experiments reported in (B) was different to that of (A), with a more extensive degree of N-terminal Met cleavage; hence, the +131 Da peak is less well resolved.


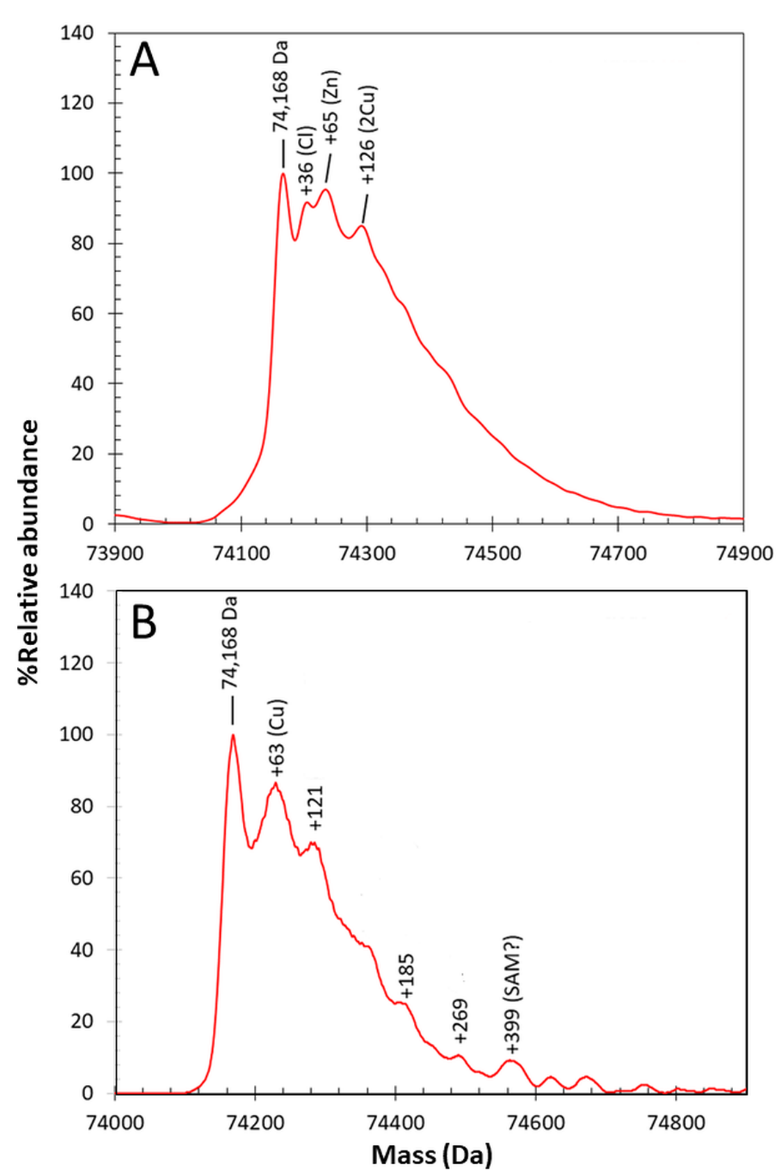


**Fig. S2. Mass spectrometric analyses of dimeric DsyB under non-denaturing conditions**. (A) Deconvoluted non-denaturing mass spectrum of as-isolated DsyB (~20 μM) in 250 mM ammonium acetate pH 8.0 buffer. (B) As in (A) but DsyB was pre-activated by addition of lysate from *L. aggregata* *dsyB* deletion strain. The mass of the main DsyB dimer peak is indicated and adduct species are labelled with the additional mass and origin (if known).


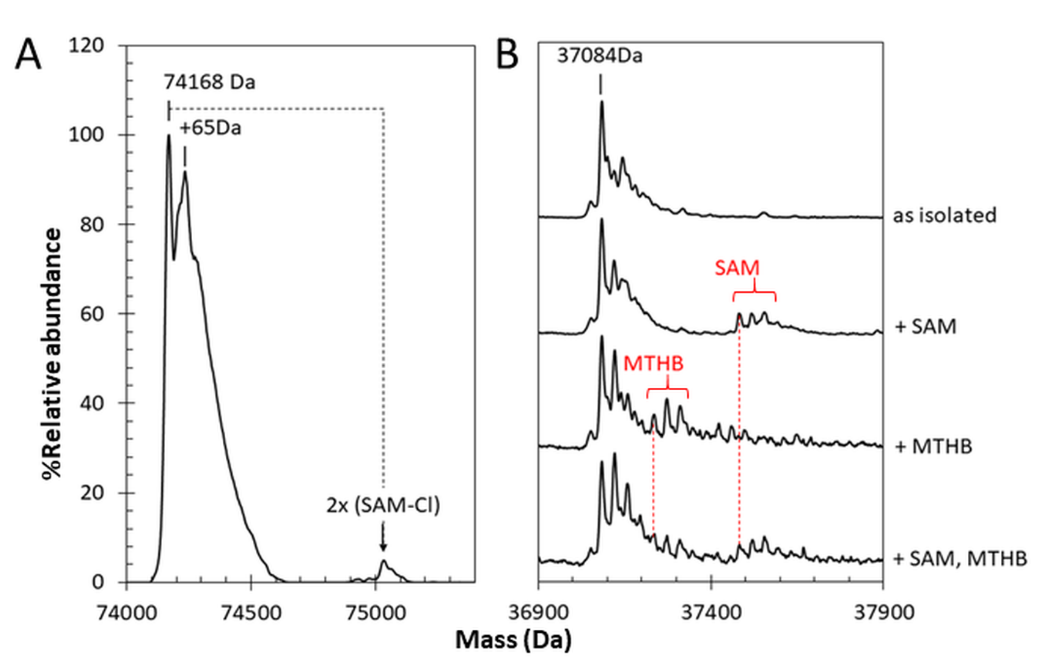


**Fig. S3. Substrate binding to DsyB probed by mass spectrometry**. Deconvoluted mass spectra of DsyB under non-denaturing conditions in the presence of SAM and MTHB. (A) Peaks due to a DsyB dimer, metal (+65 Da) and (SAM-Cl)_2_ adducts are indicated. (B) The DsyB monomer and adducts due to SAM and MTHB are as indicated. Presence of both SAM and MTHB leads to reduction in substrate adduct peaks.


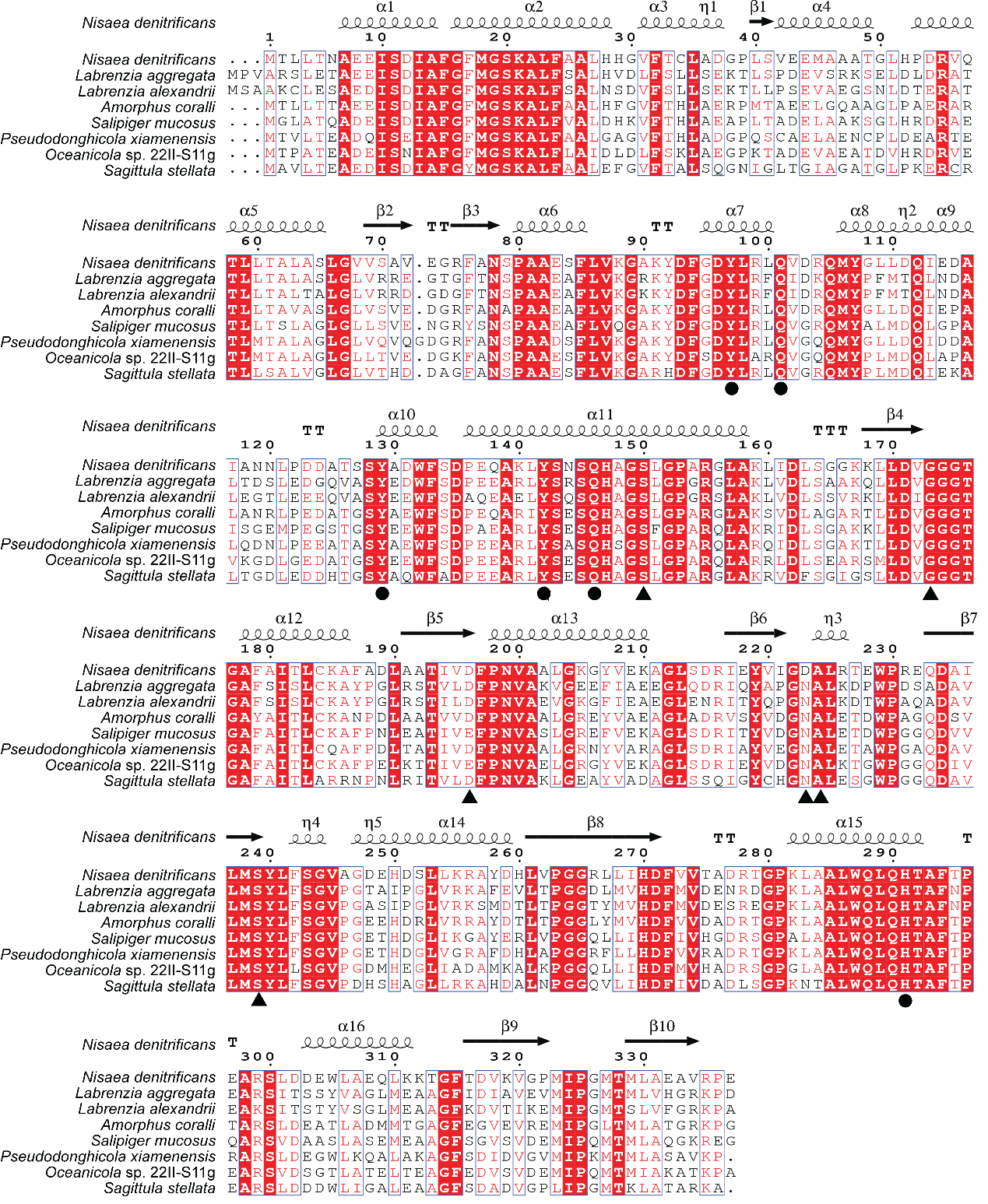


**Fig. S4. Sequence alignment of bacterial DsyB proteins.** *Nisaea denitrificans* belongs to the order Rhodospirillales, *Amorphus coralli* belongs to Rhizobiales, and the other strains are Rhodobacterales. Black dots indicate residues involved in MTHB binding, and black triangles indicate residues involved in SAM binding.


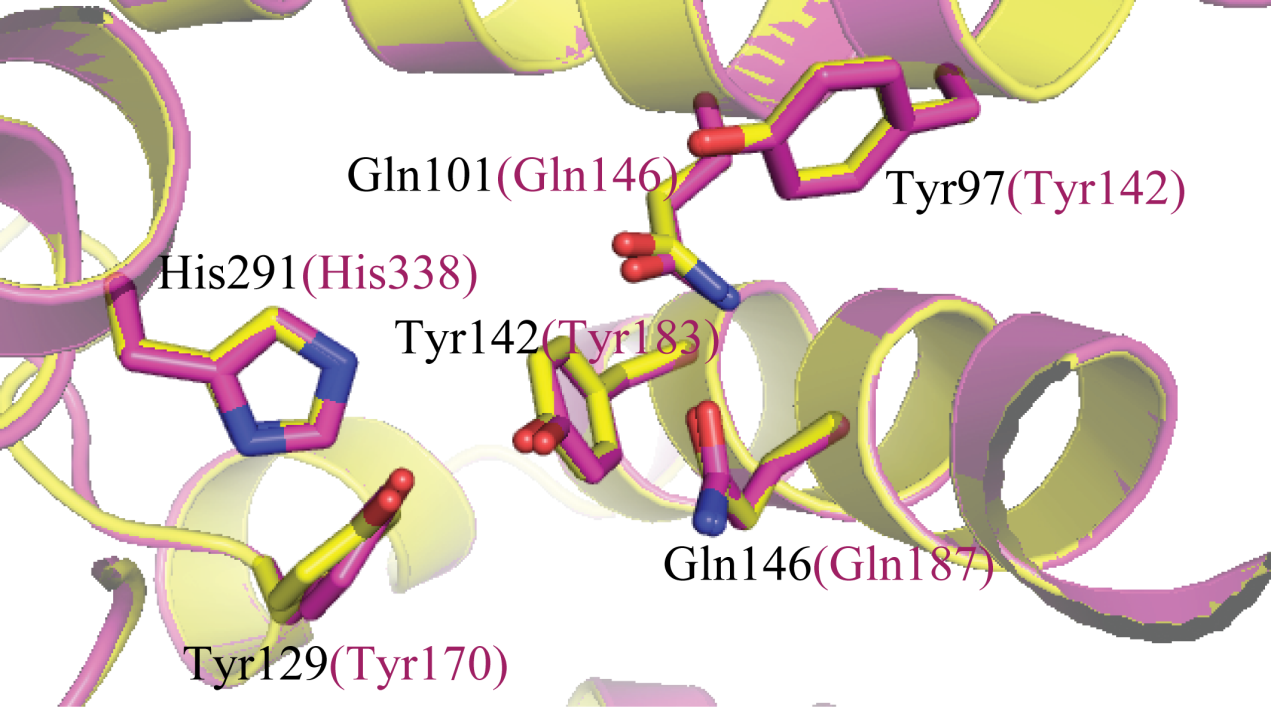


**Fig. S5. Structural alignment of DsyB from *N. denitrificans* (yellow) and DSYB from *Chrysochromulina tobin* CCMP291 (purple).** The structure of DSYB was modelled using SWISS-MODEL (https://swissmodel.expasy.org/). Residues involved in binding MTHB from DsyB and from DSYB are labelled in black and purple, respectively.


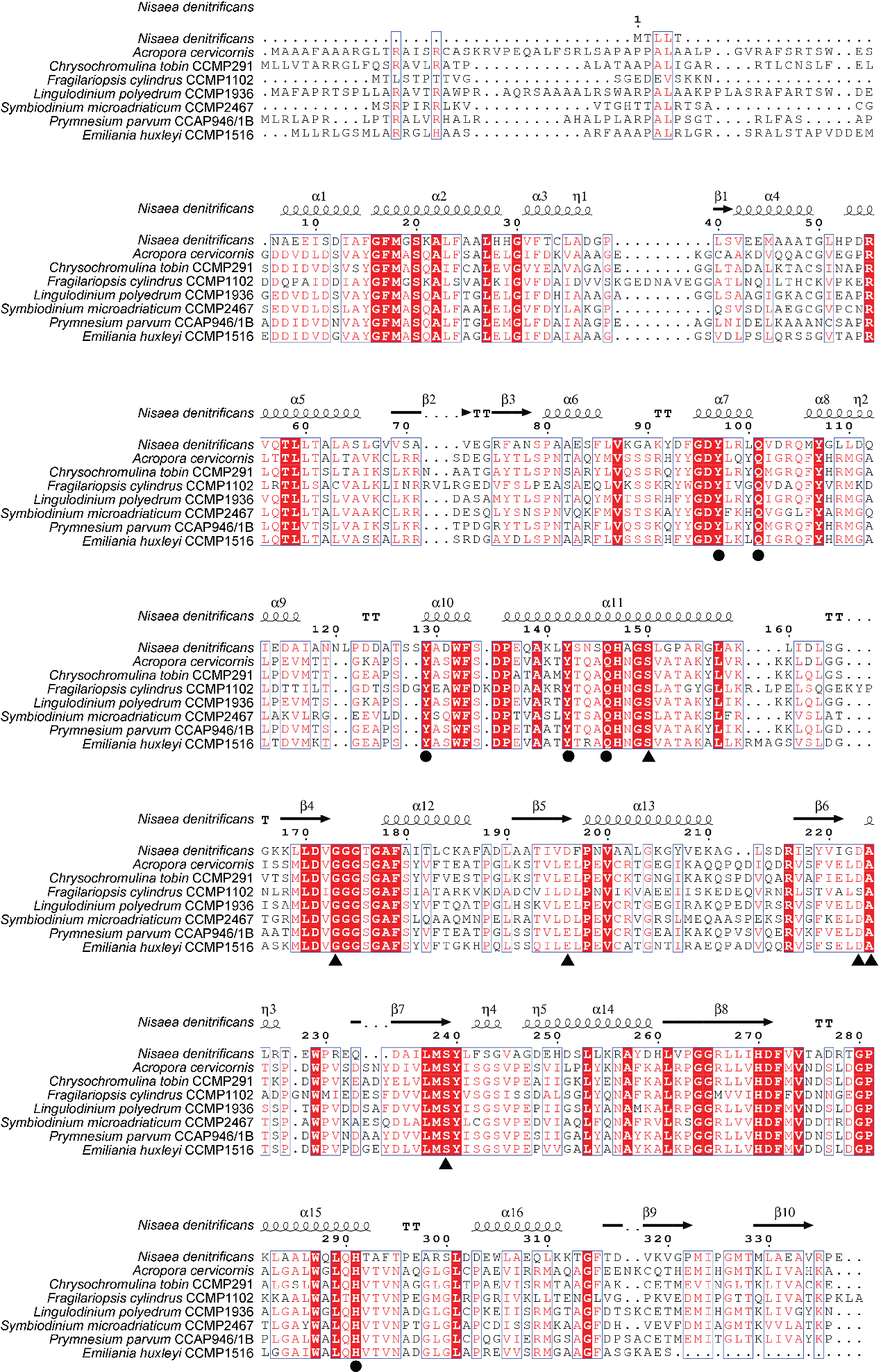


**Fig. S6.** **Sequence alignment of DsyB and eukaryotic DSYB proteins.** Black dots indicate residues involved in MTHB binding, and black triangles indicate residues involved in SAM binding.


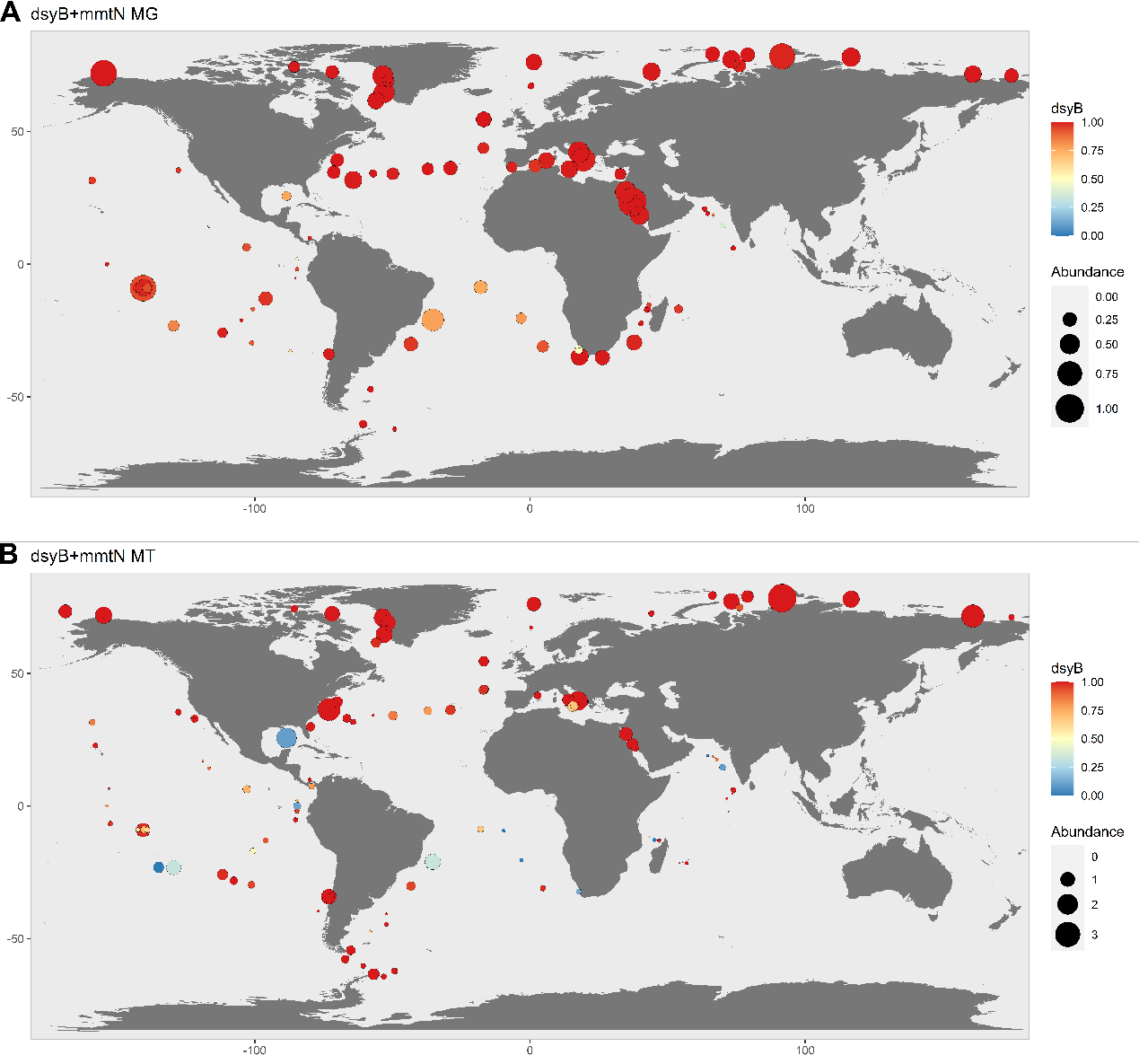


**Fig. S7.** **Bubble plot of global distribution of *dsyB/mmtN* in ocean metagenomes (A) and metatranscriptomes (B).** The sum of the average abundances of *dsyB* and *mmtN* across all depths as a percentage of the median of 10 single copy marker genes are shown by the bubbles. The colour bar indicates the relative abundance of *dsyB:mmtN* with red indicating 100% *dsyB* and blue indicating 100% *mmtN*.


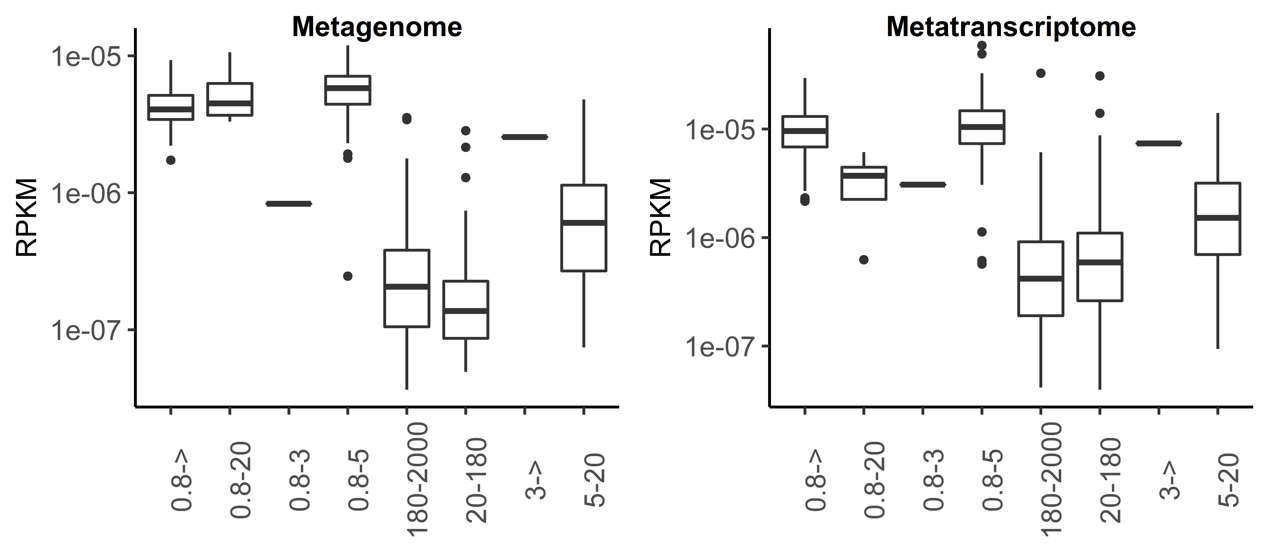


**Fig. S8.** ***DSYB* abundance (reads per kilobase per million mapped reads) by filter fraction size in the metagenome and metatranscriptome databases.**

**Table S1. Crystallographic data collection and refinement**

| Parameters | DsyB-SAM complex  Se derivative | DsyB-SAM complex | DsyB-SAH-MTHB  complex |
| --- | --- | --- | --- |
| **Diffraction data** |  |  |  |
| Space group | *P*2_1_2_1_2_1_ | *P*2_1_2_1_2_1_ | *P*2_1_ |
| Unit cell |  |  |  |
| a, b, c (Å) | 75.6, 114.9, 152.6 | 76.5, 115.9, 153.1 | 88.1, 69.3, 104.3 |
| α, β, γ (°) | 90.0, 90.0, 90.0 | 90.0, 90.0, 90. 0 | 90.0, 92.2, 90.0 |
| Resolution range (Å) | 50.0-2.7 (2.8-2.7) * | 50.0-2.4 (2.49-2.40) | 50.0-2.4 (2.49-2.40) |
| Redundancy | 26.2 (25.3) | 6.8 (7.0) | 3.7 (3.7) |
| Completeness (%) | 100.0 (100.0) | 99.9 (100.0) | 99.7 (99.7) |
| *R*_merge_** | 0.2 (0.4) | 0.1 (0.5) | 0.1 (0.5) |
| *I*/σ*I* | 37.8 (9.9) | 42.5 (6.9) | 24.0 (4.1) |
| **Refinement statistics** |  |  |  |
| R-factor |  | 0.19 | 0.19 |
| Free R-factor |  | 0.25 | 0.27 |
| RMSD from ideal geometry |  |  |  |
| Bond lengths (Å) |  | 0.008 | 0.008 |
| Bond angles (°) |  | 1.16 | 1.13 |
| Ramachandran plot (%) |  |  |  |
| Favoured |  | 93.7 | 95.9 |
| Allowed |  | 6.2 | 4.1 |
| Outliers |  | 0.1 | 0 |
| Overall B-factors (Å^2^) |  | 46.9 | 43.0 |

*Numbers in parentheses refer to data in the highest resolution shell.

** *R*_merge_=∑*_hkl_*∑*_i_*|*I*(*hkl*)*_i_* -<*I*(*hkl*)>|/∑*_hkl_*∑*_i_I*(*hkl*)*_i_*, where *I* is the observed intensity, <*I*(*hkl*)> represents the average intensity, and *I*(*hkl*)*_i_* represents the observed intensity of each unique reflection.

**Table S5. Strains and Plasmids used in this study.**

| **Strain/Plasmid** | **Description** | **Reference** |
| --- | --- | --- |
| *Escherichia coli* BL21 (DE3) | Strain used for DsyB expression for protein purification | New England BioLabs. |
| *Rhizobium leguminosarium* J391 | Streptomycin resistant derivative of wild type strain 3841 used for the expression of genes cloned in plasmid pLMB509 | Young et al. (2006) |
| *Labrenzia aggregata* J571 | *Labrenzia aggregata* LZB033 with mutation in *dsyB* gene. | Curson et al. (2017) |
| *Nisea denitrificans* DR41_21 | Wild type strain DSM 18348 | DSMZ, Leibniz Institute, Germany. |
| pLMB509 | Plasmid vector for taurine inducible expression of cloned genes in J391 and J571. | Tett et al. (2012) |
| pRK2013 | helper plasmid used in triparental mating | Figuski and Helinski. (1979) |
| SNP-1304 | *Nisaea denitrificans* DR41_21 *dsyB* gene coned into pLMB509 | this study |
| SNP-1305 | derivative of SNP-1304 with Y97A mutation | this study |
| SNP-1306 | derivative of SNP-1304 with Q101A mutation | this study |
| SNP-1307 | derivative of SNP-1304 with Y129A mutation | this study |
| SNP-1308 | derivative of SNP-1304 with Y142A mutation | this study |
| SNP-1309 | derivative of SNP-1304 with Q146A mutation | this study |
| SNP-1310 | derivative of SNP-1304 with H291A mutation | this study |

**Table S6. Primers used in this study.**

| **Primers** | **Sequence (5’-3’)** | **Purpose** |
| --- | --- | --- |
| DsyB-F | GGAATTCCATATGACGTTGCTGACAAACGCC | Amplification of the genomic *dsyB* gene |
| DsyB-R | CCGCTCGAGCTCCGGCCGCACGGCCTCG |  |
| Y97A-F | AATACGATTTCGGCGACGCTCTGCGTCTGCAGGTGG | Construction of the mutant Tyr97Ala |
| Y97A-R | CCACCTGCAGACGCAGAGCGTCGCCGAAATCGTATT |  |
| Y97F-F | CGAAATACGATTTCGGCGACTTTCTGCGTCTGC | Construction of the mutant Tyr97Phe |
| Y97F-R | GCAGACGCAGAAAGTCGCCGAAATCGTATTTCG |  |
| Q101A-F | GACTATCTGCGTCTGGCGGTGGACCGGCAGAT | Construction of the mutant Gln101Ala |
| Q101A-R | ATCTGCCGGTCCACCGCCAGACGCAGATAGTC |  |
| Q101A-F | CTATCTGCGTCTGGAGGTGGACCGGCA | Construction of the mutant Gln101Glu |
| Q101A-R | TGCCGGTCCACCTCCAGACGCAGATAG |  |
| Y129A-F | GATGCCACCAGCTCCGCCGCCGACTGGTTCTC | Construction of the mutant Tyr129Ala |
| Y129A-R | GAGAACCAGTCGGCGGCGGAGCTGGTGGCATC |  |
| Y129F-F | GATGCCACCAGCTCCTTCGCCGACTG | Construction of the mutant Tyr129Phe |
| Y129F-R | CAGTCGGCGAAGGAGCTGGTGGCATC |  |
| Y142A-F | CGGAACAGGCAAAGCTCGCTTCCAACAGCCAGCATG | Construction of the mutant Tyr142Ala |
| Y142A-R | CATGCTGGCTGTTGGAAGCGAGCTTTGCCTGTTCCG |  |
| Y142F-F | GGAACAGGCAAAGCTCTTTTCCAACAGCCAGCATG | Construction of the mutant Tyr142Phe |
| Y142F-R | CATGCTGGCTGTTGGAAAAGAGCTTTGCCTGTTCC |  |
| Q146A-F | CTCTATTCCAACAGCGCGCATGCCGGCTCCCT | Construction of the mutant Gln146Ala |
| Q146A-R | AGGGAGCCGGCATGCGCGCTGTTGGAATAGAG |  |
| Q146E-F | TCTATTCCAACAGCGAGCATGCCGGCTCC | Construction of the mutant Gln146Glu |
| Q146E-R | GGAGCCGGCATGCTCGCTGTTGGAATAGA |  |
| H291A-F | CTGGCAGCTCCAGGCCACCGCCTTCACG | Construction of the mutant His291Ala |
| H291A-R | CGTGAAGGCGGTGGCCTGGAGCTGCCAG |  |
| N.d dsyBF  N.d dsyBR  N.d gyrBF  N.d gyrBF  N.d recAF  N.d recAF | GGGTCTAAGGCGTTATTTG  CAGTCGGCGTAGGAGC  CTATCACGAAAACACGCTC  GCTTCATACGCCTTGGA  AGAATGGCGGCACTTG  CTAGGCAGCGAGACTTTG | For RT-qPCR |
|  |  |  |
